# Comparative Genomics of Sex‐Determination‐Related Genes Reveals Shared Evolutionary Patterns Between Bivalves and Mammals, but Not Fruit Flies

**DOI:** 10.1111/mec.70103

**Published:** 2025-09-21

**Authors:** Filippo Nicolini, Sergey V Nuzhdin, Fabrizio Ghiselli, Andrea Luchetti, Liliana Milani

**Affiliations:** ^1^ Department of Biological, Geological and Environmental Sciences University of Bologna Bologna Italy; ^2^ Fano Marine Center Fano Italy; ^3^ Department of Molecular and Computational Biology University of Southern California Los Angeles California USA

**Keywords:** Dmrt genes, Fox genes, molecular evolution, molluscs, reproduction, Sox genes

## Abstract

The molecular basis of sex determination (SD), while being extensively studied in model organisms, remains poorly understood in many animal groups. Bivalves, a diverse class of molluscs with a variety of reproductive modes, represent an ideal yet challenging clade for investigating SD and the evolution of sexual systems. However, the absence of a comprehensive framework has limited progress in this field, particularly regarding the study of sex‐determination‐related genes (SRGs). In this study, we performed a genome‐wide sequence evolutionary analysis of the Dmrt, Sox and Fox gene families in more than 40 bivalve species. For the first time, we provide an extensive and phylogenetically aware dataset of these SRGs, and we find support for the hypothesis that *Dmrt‐1L* and *Sox‐H* may act as primary sex‐determining genes by showing their high levels of sequence diversity within the bivalve genomic context. To validate our findings, we studied the same gene families in two well‐characterised systems, mammals and fruit flies (genus *Drosophila*). In the former, we found that the male sex‐determining gene *Sry* exhibits a pattern of amino acid sequence diversity similar to that of *Dmrt‐1L* and *Sox‐H* in bivalves, consistent with its role as master SD regulator. In contrast, no such pattern was observed among genes of the fruit fly SD cascade, which is controlled by a chromosomic mechanism. Overall, our findings highlight similarities in the sequence evolution of some mammal and bivalve SRGs, possibly driven by a comparable architecture of SD cascades. This work underscores once again the importance of employing a comparative approach when investigating understudied and non‐model systems.

**Abbreviations**
AASD: amino acid sequence divergence
DGE: differential gene expression
DM [domain]: *dsx* and *mab‐3* [domain]
Dmrt: *doublesex* and *mab‐3* related transcription factor
*Dmrt‐1L*
: *doublesex and mab‐3 related transcription factor 1‐like*
*Dm‐W*
: *doublesex and mab‐3‐related gene W*
*Dmy*
: *doublesex and mab‐3‐related gene Y*
DSFGs: Dmrt, Sox and Fox genes
*dsx*
: *doublesex*
Fox: forkhead box
ISH: in situ hybridisation
Mya: million years ago
SCO: single‐copy orthogroup
SD: sex determination
SDG: sex‐determining gene
Sox: *Sry*‐related HMG box
SRG: sex‐determination‐related gene
*Sry*
: *sex‐determining region Y*

## 1 Introduction

In sexually reproducing organisms, the mechanism of sex determination (SD), i.e., the process by which the male or female identity of an organism (or gonadic tissue) is established, is highly diverse, ranging from strictly genetic systems to environmentally dependent processes (Haag and Doty 2005; Uller and Helanterä 2011; Bachtrog et al. 2014; Beukeboom and Perrin 2014). Characterising the molecular basis of SD is crucial for understanding not only the reproductive biology but also the evolutionary pressures shaping these systems (Wilkins 1995; Ellegren and Parsch 2007; Grath and Parsch 2016; Nicolini, Ghiselli, et al. 2023), as sex‐determination‐related genes (SRGs; including primary sex‐determining genes [SDGs]) are those responsible for the phenotypic differences between males and females, thanks to their sex‐biased expression and interactions (Ellegren and Parsch 2007; Beukeboom and Perrin 2014; Grath and Parsch 2016). One key aspect of SRGs is that they often exhibit accelerated rates of sequence evolution due to their involvement in sex‐related traits and reproduction. This represents the effect of sexual selection and/or adaptation, which act on sex‐biased genes and produce highly divergent proteins at the interspecific level (Civetta and Singh 1998; Ellegren and Parsch 2007; Meisel 2011; Grath and Parsch 2016). Rapid sequence evolution is known for *Sex‐determining Region Y* (*Sry*) of therians (Pamilo and O'Neill 1997; Mawaribuchi et al. 2012), *Doublesex and mab‐3 related gene W* (*Dm‐W*) of the African clawed frog *Xenopus laevis* , and *Doublesex and mab‐3 related gene Y* (*Dmy*) of the medaka fish *Oryzias latipes* (Mawaribuchi et al. 2012), all of which are master SDGs, that is, genes whose expression is primarily responsible for the establishment of the sex of the organism. Evolution under episodic diversifying selection has been detected also in *Drosophila* for genes involved in the SD cascade (e.g., *Sex‐lethal* [*Sxl*], *transformer* [*tra*] and *doublesex* [*dsx*]), in correspondence with its establishment in the genus common ancestor (Mullon et al. 2012; Baral et al. 2019); though, rapid sequence evolution does not seem to concern extant amino acid sequences (Haerty et al. 2007; Baral et al. 2019), as they are globally evolving under purifying selection, especially in their catalytic domain (Mullon et al. 2012; Baral et al. 2019). Concerning the *dsx* genes, higher rates of nucleotide and amino acid sequence evolution can be observed for male‐specific regions if compared to female‐specific regions and dimerisation domains (Baral et al. 2019).

While SD has been extensively studied in model organisms, like the mouse *Mus musculus* , fruit flies of the genus *Drosophila*, and *Caenorhabditis elegans* roundworms, comparatively little is known about the molecular mechanisms in non‐model organisms, such as aquatic invertebrates. A remarkable example is represented by bivalve molluscs, which exhibit a wide variety of reproductive modes and sexual systems (Breton et al. 2018). Notwithstanding the considerable importance in the human socio‐economic landscape (reviewed in Haszprunar and Wanninger 2012; Gomes‐dos‐Santos et al. 2020), the study of SD mechanisms in bivalves has been hampered by the striking divergence among species (Li et al. 2022) and thus largely overlooked and limited to few case studies (Breton et al. 2018; Nicolini, Ghiselli, et al. 2023). So far, no master SDG has been unambiguously identified, and the only working hypothesis on the functioning of the SD gene regulatory network is available for the Pacific oyster *Magallana* (formerly *Crassostrea*) *gigas* (Zhang et al. 2014). Nonetheless, the field still lacks both a robust functional investigation and an evolutionary framework in which to place the current knowledge (Nicolini, Ghiselli, et al. 2023). As a matter of fact, major efforts have been dedicated to identifying sex‐biased genes through differential gene expression (DGE) analyses (e.g., Ghiselli et al. 2012; Milani et al. 2013; Teaniniuraitemoana et al. 2014; Zhang et al. 2014, 2025; Capt et al. 2018; Afonso et al. 2019), but very few have leveraged cutting‐edge techniques to investigate their actual role in SD and/or gonad differentiation and development (e.g., Liang et al. 2019; Sun et al. 2022).

Components of the *doublesex* and *mab‐3* related transcription factor (Dmrt), *Sry*‐related HMG box (Sox) and forkhead box (Fox) gene families (henceforth referred to as DSFG) are notoriously known as key actors in several developmental processes across Metazoa (Benayoun et al. 2011; Matson and Zarkower 2012; Sarkar and Hochedlinger 2013; Mawaribuchi et al. 2019), including SD in certain clades: the aforementioned *Dm‐W*, *Dmy* and *dsx* all belong to the Dmrt gene family, while *Sry* belongs to the Sox gene family; *Fox‐L2*, which takes part in most of the vertebrate SD processes as a downstream effector of the female pathway, belongs to the Fox gene family. Members of the DSFGs have been identified as putative SRGs also in bivalves, thanks to both DGE analyses and in situ hybridisation (ISH; e.g., Naimi et al. 2009; Liu et al. 2012; Li et al. 2018; Liang et al. 2019; Yue et al. 2021), suggesting that their role in morphological and sexual development is maintained also in the clade. However, the clear role of DSFGs has yet to be elucidated, probably as a consequence of the lack of (i) a systematic classification of the gene families and (ii) a comprehensive understanding of their evolutionary history.

To overcome such limitations, this study aims to perform a thorough investigation of the DSFG families in bivalves, with the attempt to provide a high‐quality resource to be used as a reference for future studies. Through the analysis of more than 40 annotated bivalve genomes and transcriptomes, we aim (i) to describe the complete set and evolutionary history of DSFGs in bivalves by means of phylogenetic inferences, manual curation and orthology prediction; and (ii) to identify DSFGs potentially involved in bivalve SD by investigating their sequence evolution in a genome‐wide context. Our hypothesis is that, if any of the DSFGs is directly involved in SD (i.e., is an SDG), then we should expect it to show a higher rate of sequence evolution, as already found in previous studies (Pamilo and O'Neill 1997; Mawaribuchi et al. 2012) and discussed earlier; this characteristic, in turn, would be reflected in a high diversity of the extant amino acid sequences across the bivalve clade. To assess the robustness and reliability of our approach, we additionally applied our pipeline to two non‐bivalve datasets, composed of mammal and *Drosophila* species, respectively (hereon referred to as the ‘mammal dataset’ and the ‘fruit fly dataset’). By choosing two clades for which SD is well characterised, we wanted to compare our results with those obtained on taxa for which a deeper and more detailed knowledge is available. In particular, mammals and fruit flies provide two different frameworks to study the patterns of molecular evolution in SDGs: the former is a system where SD is completely genetic (i.e., the development into a male or into a female is triggered by the up‐ or downregulation of *Sry* in undifferentiated gonads, respectively), and for which a high rate of sequence evolution is already known for the male SDG *Sry*; the latter is instead a system where SD is chromosomic, thus lacks a master SDG (the sexual fate of the individual is determined by the ratio between autosomes and X chromosomes), and no high rate of sequence evolution has been detected in extant amino acid sequences of SRGs (which are globally evolving under purifying selection). In this sense, the mammal and the fruit fly datasets represent opposing control datasets to be compared to bivalves, as it is expected that a higher rate of sequence evolution concerns only master SDGs (like *Sry* in therians; i.e., the top regulatory part of the SD cascade), but not the downstream genes (i.e., the bottom effectors). Therefore, we tested our pipeline on mammals and fruit flies with the following expectations: (i) in the mammalian dataset (which works as a positive control), *Sry* should be detected as fast‐evolving (Pamilo and O'Neill 1997; Mawaribuchi et al. 2012); while (ii) in the fruit fly dataset (which works as a negative control), no gene among those working within the sex‐determining cascade should result in evolving at a higher pace (Haerty et al. 2007; Mullon et al. 2012; Baral et al. 2019). In this way, we are able to assess the reliability of results in bivalves by comparing them to a null hypothesis.

This work offers novel insights into the evolutionary dynamics of SRGs and contributes a valuable genomic resource for understanding SD in bivalves, one of the most ecologically and economically important groups of marine organisms. In particular, here we provide the first extensive phylogenetic‐based classification of DSFGs in bivalves, covering many species from the major bivalve orders, along with a comprehensive investigation of their sequence evolution.

## 2 Materials and Methods

### 2.1 Dataset of Bivalve Annotated Genomes and Transcriptomes

Annotated genome assemblies of bivalves, gastropods and cephalopods were obtained from various publicly available resources (Table S1). Isoforms from genome annotations were removed using the AGAT toolkit (v0.8.0; Dainat n.d.). The nucleotide coding sequences for *Sinonovacula constricta* (Adapedonta) were not available for download. To avoid excluding the species from our analyses, the corresponding file was generated in‐house by mapping the annotated protein sequences on the reference genome using miniprot (v0.13‐0; Li 2023). In order to provide an extensive identification of SRGs also for under‐represented bivalve orders (mainly belonging to the Heterodonta clade), 14 additional species represented by sequenced transcriptomes were included in the analyses and were obtained from Piccinini et al. (2021), Iannello et al. (2023), and Iannello et al. 2025. The resulting set of annotated genomes and transcriptomes (hereafter referred to as the ‘comprehensive bivalve dataset’) was checked for completeness using BUSCO with the Metazoa reference dataset (v5.2.2; Manni et al. 2021).

### 2.2 Identification and Classification of Dmrt, Sox and Fox Genes in Bivalves

Members of DSFG families were retrieved from the comprehensive bivalve dataset with hmmsearch (v3.3.2; http://hmmer.org/). The signature catalytic domains of the DSFG families were used as queries. Specifically, HMM profiles were built after the Pfam databases for the DM domain (PF00751), the HMG box (PF00505) and the forkhead domain (PF00250), respectively. Obtained hits were then annotated using (i) the PANTHER HMM standalone sequence scoring against the PANTHER library v18.0 and (ii) RPS‐BLAST (v2.5.0+) against CDD (pre‐compiled version, downloaded on 09/11/23). In both cases, hits with an *E*‐value of 10^−5^ were retained. Genes that were correctly annotated by both systems (Table S2) were kept for subsequent analyses. DSFGs from *Homo sapiens* , *Drosophila melanogaster* and *C. elegans* (Table S3; the reference species) were retrieved from NCBI and were used as reference genes for annotation (see below). Classification and nomenclature of each family were retrieved from Mawaribuchi et al. (2019) for Dmrt genes; Phochanukul and Russell (2010) and Sarkar and Hochedlinger (2013) for Sox genes; and Mazet et al. (2003) for Fox genes. The alignments of mollusc and reference DSFGs were guided by the Pfam HMM profiles and performed with Clustal Omega (v1.2.3; Sievers et al. 2011), then trimmed with trimAl (v1.4.rev15; Capella‐Gutiérrez et al. 2009) with a gap threshold of 40%. Resulting alignments were manually inspected to remove sequences with incomplete catalytic domains, then aligned and trimmed again as before. Phylogenetic trees were inferred using IQ‐TREE (v2.1.4‐beta COVID‐edition; Minh et al. 2020) with automatic model selection (Kalyaanamoorthy et al. 2017) and 1000 bootstrap replicates. The phylogenetic tree of Dmrt genes was midpoint rooted, as no clear outgroup has been found so far (Wexler et al. 2014). Phylogenetic trees of Sox and Fox gene families were rooted using two fungi MATA‐1 sequences (XP_62685912.1, CCD57795.1) and two Amoebozoa forkhead‐like domains (XP_004368148.1, XP_004333268.1), respectively (Heenan et al. 2016; Nakagawa et al. 2013). The rooting was performed with Gotree (v0.4.5; Lemoine and Gascuel 2021). To identify and annotate bivalve homology groups within each gene family, we utilised the algorithm implemented in Possvm (v1.2; Grau‐Bové and Sebé‐Pedrós 2021), with DSFGs from the reference species as reference annotation. To better establish the orthology relationships among ambiguous groups of Dmrt and Fox genes, we ran a series of other phylogenetic reconstructions (see Discussion) by using the same pipeline as before. In the case of *Fox‐Y* genes, we also used Fox gene sequences from the sea urchin *Strongylocentrotus purpuratus* , as given by Tu et al. (2006). All the phylogenetic trees were plotted using TreeViewer v2.2.0 (Bianchini and Sánchez‐Baracaldo 2024).

### 2.3 Sequence Diversity of Bivalve Single‐Copy Orthogroups

**FIGURE 1:**
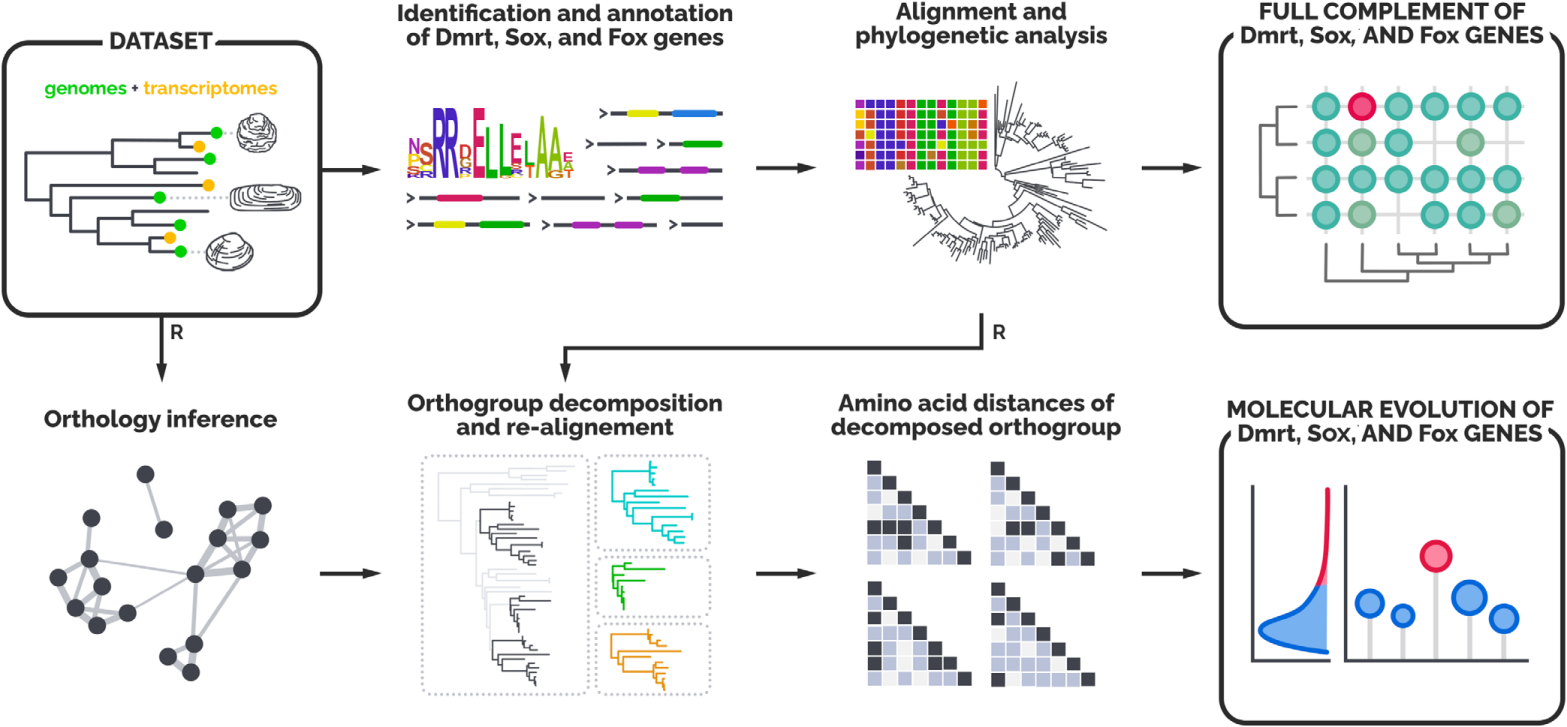
Workflow for the bivalve dataset. Starting from a set of both genomes and transcriptomes covering a great portion of bivalve taxonomic diversity, we first characterised the entire complement of Dmrt, Sox and Fox genes (upper row). We used sequence annotation and phylogenetic tools to obtain reliable sequences and to filter out any putative misassembled or misannotated sequences. Afterwards, we built a reduced set of transcriptomes and genomes (the reduced bivalve dataset, where we minimised the redundancy of congeneric species) from which to draw the molecular evolution patterns of orthologous genes (bottom row). After having obtained single‐copy orthologous groups, we calculated the amino acid distances within each orthogroup and then built the distribution of median values. The same pipeline was also utilised for the mammal and the fruit fly datasets, with just two minor differences: the starting dataset was composed of only genomes, and the reduction step (R) was not necessary.

As a metric to measure the sequence diversity of bivalve DSFGs and test whether those putatively involved in SD showed higher values than other genes, we used the amino acid sequence divergence (AASD). This metric is fast and straightforward to obtain, as it only requires the amino acid alignment and the corresponding best‐fit substitution model. To this purpose, we produced amino acid alignments of bivalve single‐copy orthogorups (SCOs), and built the distribution of their median AASD. To further save computational time and prevent over‐represented bivalve clades (such as Ostreida and Mytilia) from biasing the analysis, SCOs were obtained after a reduced dataset comprised of 34 bivalve species (hereafter referred to as the ‘reduced bivalve dataset’; Figure 1; Table S1), which included, for each bivalve genus, only the best genomes and transcriptomes in terms of either BUSCO scores or assembly statistics (Table S1). *Archivesica marissinica* and *Saccostrea glomerata* were removed, as their annotated coding sequences contain many stop codons. Genes were clustered in orthogroups using OrthoFinder (v2.5.5; Emms and Kelly 2019) with DIAMOND ultra‐sensitive (Buchfink et al. 2015) and default parameters. Resulting orthogroups were split into SCOs composed of at least 17 species (50% of the bivalve reduced dataset) using DISCO (v1.3.1; Willson et al. 2022). Amino acid and nucleotide sequences were then aligned using Clustal Omega as implemented in TranslatorX (v1.1; Abascal et al. 2010) and jointly trimmed using trimAl with a gap threshold of 40% and the removal of spurious sequences (‐resoerlap 50 ‐seqoverlap 50). Eventually, SCOs containing (i) internal stop codons, (ii) with less than 17 species left, or (iii) containing DSFGs were removed from downstream analyses. The best amino acid substitution model was inferred for each trimmed alignment using ModelFinder as implemented in IQ‐TREE2 (model search was restricted to the following matrices: Blosum62, cpREV, Dayhoff, DCMut, FLU, HIVb, HIVw, JTT, JTTDCMut, LG, mtART, mtMAM, mtREV, mtZOA, rtREV, VT, WAG), and the corresponding pairwise amino acid distances were computed with the function ‘dist.ml’ from the ‘phangorn’ R package (Schliep 2011). We decided to use the pairwise amino acid distance instead of the tip‐to‐tip phylogenetic distance (which accounts for a more comprehensive evolutionary signal) to save computational time. However, to check whether the two metrics were comparable to each other, we randomly selected 200 SCOs and computed the maximum likelihood (ML) trees using IQ‐TREE2, with ModelSelection restricted as before. Then, the tip‐to‐tip pairwise distances were obtained with the R package ‘adephylo’ (Jombart and Dray 2010). The same pipeline to obtain SCOs and AASD was also utilised on the DSFG families. The distribution of AASD was then built after the median values of pairwise distances of each SCO, and genes were categorised accordingly into three groups: Group 1, consisting of genes from the 1% upper quantile of the distribution; Group 2, consisting of genes between the 1% and 5% upper quantiles; and Group 3, consisting of all the remaining genes. Group 1 and Group 2 genes will be referred to as ‘highly divergent genes’.

### 2.4 Test for Positive or Relaxed Selection

High AASD of genes involved in SD and/or in reproductive processes has been hypothesised to be driven by either sexual or positive (diversifying) selection (Swanson and Vacquier 2002; Vicoso and Charlesworth 2006; Meisel and Connallon 2013; Parsch and Ellegren 2013; Grath and Parsch 2016). Recently, a different scenario has been emerging, that is, SRGs and genes involved in reproduction may be instead shaped by relaxed selection (Barker et al. 2005; Harrison et al. 2015; Dapper and Wade 2016, 2020), due to sex‐biased or sex‐specific expressions. The effect of relaxed selection on the rate of non‐synonymous substitutions over synonymous substitutions is similar to that produced by positive selection (Dapper and Wade 2020). Therefore, we assessed whether SRGs with high AASD have been shaped by positive selection or relaxed selection. The former was tested using BUSTED—which detects whether a gene has experienced positive selection at least in one site and in one branch (v3; Murrell et al. 2015)—while the latter was tested with RELAX—which detects whether natural selection has relaxed or intensified along a group of branches (v3; Wertheim et al. 2015)—both from the HyPhy package (v2.5.8; Kosakovsky Pond et al. 2020). ML phylogenetic trees were produced with IQ‐TREE for each DSFGs from the previously generated trimmed codon alignments (see Sequence Diversity Of Bivalve Single‐Copy Orthogroups). The best nucleotide substitution model was inferred with ModelFinder. Terminal branches were tested on both BUSTED and RELAX, while internal branches were set to be the background.

### 2.5 Mammals and Fruit Flies as Test Datasets

To validate our approach for the study of bivalve SRG molecular evolution, we run the same analysis on two additional datasets, consisting of reference genomes of mammals and fruit flies (Tables S4 and S5 respectively), whose sex‐determining mechanisms are well studied and characterised. As a matter of fact, despite it being well known that SDGs tend to evolve faster than genes not involved in SD, the hypothesis has never been tested extensively across the entire phylogenetic diversity of a group: molecular evolution of SDGs and SRGs has mainly been tested on single species or inside the boundaries of taxonomic genera (Stothard and Pilgrim 2003; Haerty et al. 2007; Mank et al. 2007; Mullon et al. 2012; Papa et al. 2017; Ghiselli et al. 2018). For both mammals and fruit flies, annotated genomes were downloaded from NCBI using the command‐line tool ‘datasets’, then processed using the same pipeline and scripts as before (Figure 1).

### 2.6 GO Term Enrichment

After having obtained the distributions of AASD in the three datasets (bivalves, mammals and fruit flies), we performed a gene ontology (GO) enrichment analysis of highly divergent genes. To do so, we firstly selected one gene per SCO, giving priority to a few chosen species: (i) for bivalves, we selected genes from *Pecten maximus* , or alternatively from *Crassostrea gigas* , *Hyriopsis bialata* , *Tridacna squamosa*, and *Solen grandis*; (ii) for mammals, we selected genes from *H. sapiens* , or alternatively from *Bubalus bubalis* , *Panthera tigris* , *Camelus dromedarius* , and *Monodelphis domestica* ; (iii) for fruit flies, we selected genes from *D. melanogaster* , or alternatively from *Drosophila hydei* , *Drosophila pseudoobscura* and *Drosophila suzukii*. Afterwards, we annotated the obtained datasets with the corresponding GO terms using the OMA browser (accessed 18/09/2024; Altenhoff et al. 2024). The GO‐term enrichment was then performed with the R package ‘topGO’ using the Fisher's exact test (Alexa and Rahnenfuhrer 2024) and the ’elim’ algorithm (Alexa et al. 2006).

## 3 Results

### 3.1 Assembly of the Bivalve, Mammal and Fruit Fly Datasets

The comprehensive bivalve dataset from which we retrieved and analysed DSFGs consists of 29 bivalve genomes, 14 bivalve transcriptomes and 7 outgroup genomes (5 gastropods and 2 *Octopus* spp.; Table S1). BUSCO statistics for complete single‐copy genes spanned from 64.9% in *Modiolus modiolus* to 99.4% for *Perna viridis*, with a median value of 94.7%. We were able to get at least one representative species for 11 different bivalve orders, covering a good proportion of the phylogenetic diversity of the clades Pteriomorphia, Palaeoheterodonta and Imparidentia, and thus building one of the most extensive genomic and transcriptomic dataset for bivalve comparative analyses so far. Unfortunately, no genomes or transcriptomes for Protobranchia, Archiheterodonta and Anomalodesmata were available at the time of the project; thus, we were not able to include any of those clades in our analysis. The mammal dataset consists of 32 species and 1 outgroup ( *Gallus gallus* , Aves; Table S4) and covers 12 major orders, while the fruit fly dataset consists of 17 species and 1 outgroup ( *Anopheles gambiae* , Culicidae; Table S5) and covers 2 *Drosophila* subgenera (i.e., *Drosophila* and *Sophophora*). BUSCO statistics for complete single‐copy genes were generally higher than those of bivalves, with a median of 98.3% for mammals and 99.8% for fruit flies (Tables S4 and S5).

### 3.2 Dmrt, Sox and Fox Complements in Bivalves

**FIGURE 2:**
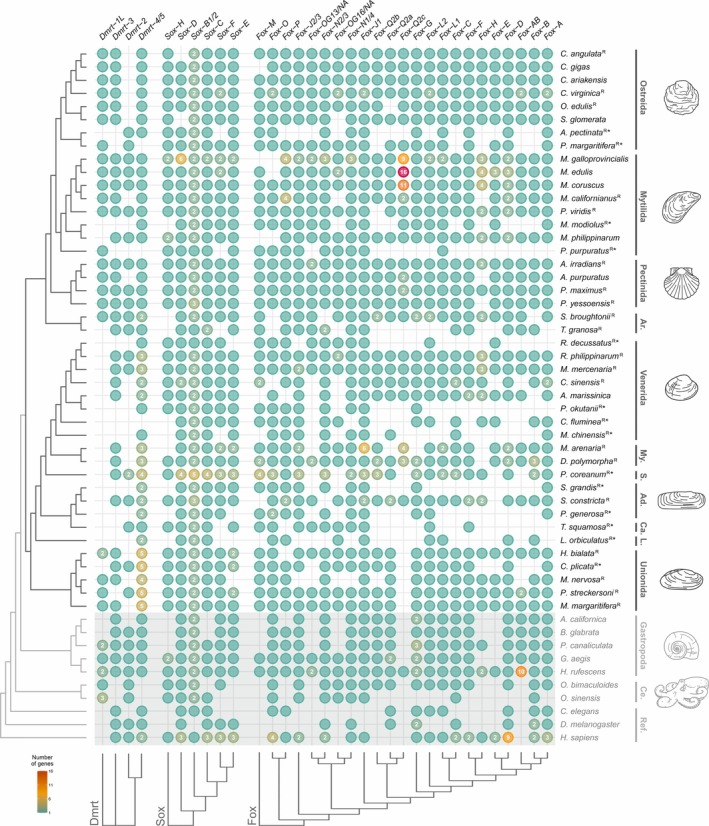
Dmrt, Sox and Fox gene (DSFG) complement in bivalves and their outgroups. The presence of genes is indicated by filled circles. Numbers inside each circle specify genes with two or more copies. The shaded area highlights non‐bivalve species, belonging either to other molluscs or to the references. The phylogenetic tree of analysed species, as inferred from literature, is shown on the left, while major taxonomic groups are reported on the right. Species present in the reduced bivalve dataset are marked with an ‘R’ (see main text and Figure 1), and species represented by transcriptomic data are marked with an asterisk (‘*’). DSFG trees are shown on the bottom; note that the Dmrt gene tree is unrooted, as at the moment no current outgroup is known for the gene family (full trees can be found in Figures S1–, S3). Full species names, along with all assembly and taxonomic information, can be found in Table S1. Ad.: Adapedonta; Ar.: Arcida; Ca.: Cardiida; Ce.: Cephalopoda; L.: Lucinida; My.: Myida; Ref.: reference genes; S.: Sphaeriida. Trees were plotted using the R package ‘ggtree’ (Yu, Smith, et al. 2017).

Our pipeline managed to successfully identify and annotate DSFGs in bivalves, as proved by the same analysis in mammals and fruit flies (see following sections). We retrieved four main orthology groups of Dmrt genes in bivalves (Figures 2 and S1; Table S6), three corresponding to the groups present in the Bilateria common ancestor (*Dmrt‐2*, *Dmrt‐3* and *Dmrt‐4/5*; Mawaribuchi et al. 2019), and one additional group with no clear ortholog among reference genes, and thus putatively specific to molluscs (named *Dmrt‐1L*, as per Li et al. 2018; Evensen et al. 2022). The *Dmrt‐4/5* subgroup shows a group‐specific expansion in Palaeoheterodonta and Heterodonta, while *Dmrt‐1L* is completely absent from Heterodonta. The ubiquitin‐binding CUE‐like DMA domain has been annotated in most of the *Dmrt‐3* and *Dmrt‐4/5* genes, while an additional DM domain has been annotated in *Dmrt‐1L* genes in Mytilida and the gastropod *Pomacea canaliculata* (Table S6). For Sox genes, we retrieved six main orthology groups, none of which is restricted to molluscs or bivalves (Figures 2 and S2; Table S6). Five Sox groups (*Sox‐B1/2*, *Sox‐C*, *Sox‐D*, *Sox‐E* and *Sox‐F*) are those traditionally considered to be present in the Bilateria common ancestor (Phochanukul and Russell 2010), while one has been identified outside mammals only recently (*Sox‐H*, or *Sox‐30*; Han et al. 2010). *Sox‐B2* and *Sox‐B1* have been grouped in the same clade, as in our phylogenetic reconstruction *Sox‐B2* is paraphyletic with respect to *Sox‐B1* (Figure S2), despite being traditionally recognised as a separate paralogy group in humans, fruit flies and roundworms. The Sox N‐terminal signature domain was annotated for *Sox‐E* genes (Table S6). Concerning Fox genes, we retrieved 27 main orthology groups (Figures 2 and S3; Table S6), two of which are specific to molluscs (*Fox‐OG13/NA*, *Fox‐OG16/NA*). Additionally, other potential mollusc‐specific Fox groups have been identified, but these have been excluded from the final orthology analysis as they are present in less than half of bivalve species (Table S6). The two major Fox gene subgroups, Group I (monophyletic, specific to Metazoa; includes *Fox‐A*, *Fox‐B*, *Fox‐C*, *Fox‐D*, *Fox‐E*, *Fox‐F*, *Fox‐G*, *Fox‐H*, *Fox‐L1*, *Fox‐L2*, *Fox‐Q2*) and Group II (paraphyletic, specific to Opisthokonta; includes *Fox‐O*, *Fox‐P*, *Fox‐J2*, *Fox‐J1*, *Fox‐K*, *Fox‐N2/3*, *Fox‐N1/4*; Larroux et al. 2008), have been recovered, including the four Fox genes that were present in the Bilateria common ancestor (*Fox‐C*, *Fox‐F*, *Fox‐L1* and *Fox‐Q1*; Shimeld et al. 2010). Two putative lineage‐specific expansions have been recovered for *Fox‐Q2c*, one regarding *Mytilus* spp. and one regarding the two Myida species (Figures 2 and S3). The forkhead‐associated (FHA) domain was annotated for *Fox‐K* genes, the *Fox‐P* coiled‐coil signature domain was annotated for *Fox‐P* genes, while both the forkhead N‐ and C‐terminal signature domains were annotated for *Fox‐A* genes (Table S6).

### 3.3 Amino Acid Sequence Divergence of Dmrt, Sox and Fox Genes in Bivalves

**FIGURE 3:**
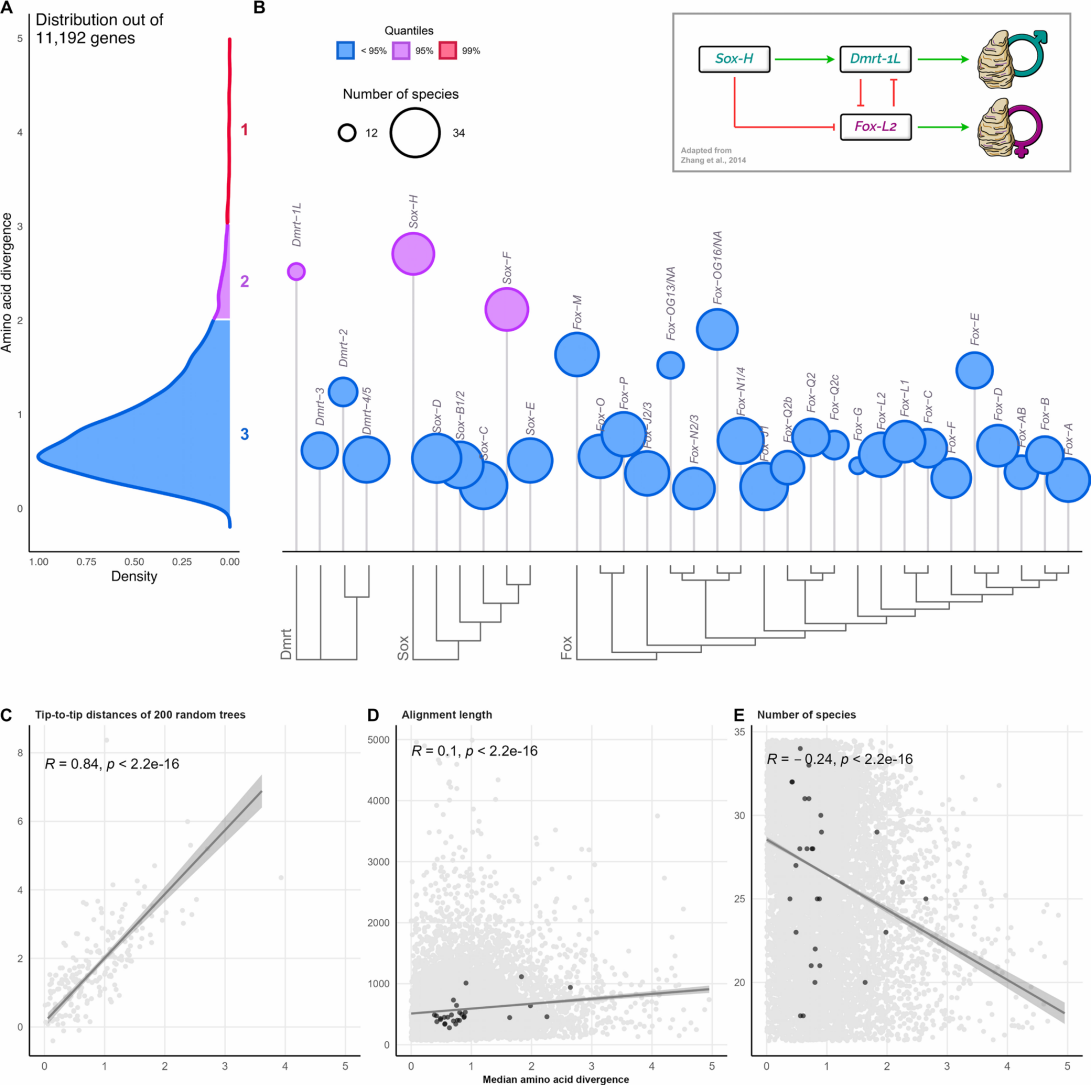
Distribution of amino acid sequence divergence (AASD) of single‐copy orthogroups in bivalves (A), including Dmrt, Sox and Fox genes (DSFGs; B), and their correlations with tip‐to‐tip distances (C), alignment lengths (D) and number of species (E). The distribution of AASD has been computed on the median values of pairwise distances of over 11 k SCOs from the reduced bivalve dataset (see main text and Figure 1). Genes have been divided according to their median AASD value into three different groups, which are indicated by different colours and increasing numbers (Groups 1, 2 and 3). Genes from Group 1 and Group 2 are collectively named ‘highly divergent genes’. Circle heights of DSFGs show the median value of their AASD, while the size indicates the number of represented species. DSFG trees are shown on the bottom; note that the Dmrt gene tree is unrooted, as at the moment no current outgroup is known for the gene family (full trees can be found in Figures S1–, S3). Darker points in panels (C–E) indicate Dmrt, Sox and Fox gene SCOs. The correlation between the amino acid distance and the tip‐to‐tip distance has been computed on 200 randomly selected orthogroups. Insets: Scheme of the sex‐determination working model in *Crassostrea gigas* as hypothesised by Zhang et al. (2014), showing the main genes involved. Green arrows indicate transcription activations, red arrows indicate transcription suppressions. Trees were plotted using the R package ‘ggtree’ (Yu, Smith, et al. 2017).

We produced amino acid alignments of ~11 k SCOs, 32 of which belong to the DSFG families. Of these, 112 were assigned to Group 1 (1% upper quantile), 448 to Group 2 (5% upper quantile), and 10,632 to Group 3. Most of the DSFGs (29/32) fell in Group 3 (Figure 3), which means they have a median AASD comparable to the vast majority of other genes in bivalves (median level of the genomes). Just *Dmrt‐1L*, *Sox‐H* and *Sox‐F* showed higher divergences and have been accordingly placed in Group 2. Overall, pairwise AASD proved to be a good approximation of the tip‐to‐tip distances calculated on the corresponding ML phylogenetic trees (*R* = 0.84, *p* < 2.2E−16, considering 200 randomly selected trees; Figure 3C), and it also shows no influence from the alignment length (*R* = 0.11) or the number of represented species (*R* = −0.23; Figure 3D,E). Genes from Group 1 and Group 2 are strongly involved in cellular regulatory processes (such as those related to the metabolism of nucleic acids, proteins and other macromolecules), but also in development and response to external stimuli, as shown by the GO‐term enrichment analysis (Tables 1 and S7). Similar results can be drawn also when employing a dataset composed only of high‐quality genomes (BUSCO single‐copy genes > 90%), which yields ~17 k SCOs for the AASD analysis (Figure S4).

**TABLE 1:** Top enriched GO terms for highly divergent genes of bivalves, mammals and fruit flies of the genus *Drosophila*.

Dataset	GO ID	Term	Annotated genes	Significant genes	Corrected *p*‐value
Bivalvia	GO:0060255	Regulation of macromolecule metabolic process	737	59	0.04525
	GO:0080090	Regulation of primary metabolic process	673	53	0.01818
	GO:0019219	Regulation of nucleobase‐containing compound metabolic process	541	41	0.02388
	GO:0006351	DNA‐templated transcription	571	39	0.03767
	GO:0032774	RNA biosynthetic process	579	39	0.04490
	GO:0051252	Regulation of RNA metabolic process	517	37	0.02719
	GO:0006355	Regulation of DNA‐templated transcription	490	35	0.03751
	GO:2001141	Regulation of RNA biosynthetic process	491	35	0.03844
	GO:0006950	Response to stress	370	33	0.01949
	GO:0032502	Developmental process	261	27	0.04445
	GO:0006468	Protein phosphorylation	345	23	0.02483
	GO:0031325	Positive regulation of cellular metabolic process	125	17	0.00801
	GO:0010604	Positive regulation of macromolecule metabolic process	151	17	0.04047
	GO:0051172	Negative regulation of nitrogen compound metabolic process	117	16	0.00814
	GO:0051173	Positive regulation of nitrogen compound metabolic process	137	15	0.02454
	GO:0006310	DNA recombination	66	14	0.00087
	GO:0048513	Animal organ development	83	12	0.04088
	GO:0010629	Negative regulation of gene expression	78	11	0.00048
	GO:0023051	Regulation of signalling	133	11	0.02872
	GO:0045934	Negative regulation of nucleobase‐containing compound metabolic process	64	11	0.03637
	GO:0009605	Response to external stimulus	90	11	0.04544
	GO:0044419	Biological process involved in interspecies interaction between organisms	63	11	0.04761
Mammalia	GO:0006955	Immune response	1297	145	0.00061
	GO:0098542	Defence response to other organism	853	112	0.02066
	GO:0045087	Innate immune response	647	82	8.5e‐10
	GO:0001817	Regulation of cytokine production	630	51	0.04660
	GO:0042742	Defence response to bacterium	233	45	1.7e‐07
	GO:0006954	Inflammatory response	642	45	0.01735
	GO:0019221	Cytokine‐mediated signalling pathway	382	44	3.9e‐07
	GO:0002250	Adaptive immune response	342	44	1.3e‐05
	GO:0001819	Positive regulation of cytokine production	402	41	0.02723
	GO:0002697	Regulation of immune effector process	308	37	0.04426
	GO:0042110	T cell activation	432	35	0.02564
	GO:0051607	Defence response to virus	257	34	1.9e‐07
	GO:0048232	Male gamete generation	491	32	0.02255
	GO:0007283	Spermatogenesis	478	31	0.02801
	GO:0070661	Leukocyte proliferation	273	29	0.01285
	GO:0002449	Lymphocyte mediated immunity	221	29	0.04833
	GO:0070663	Regulation of leukocyte proliferation	212	25	0.01870
	GO:0050727	Regulation of inflammatory response	300	24	0.00235
	GO:0031349	Positive regulation of defence response	240	24	0.01239
	GO:0002768	Immune response‐regulating cell surface receptor signalling pathway	177	22	0.00336
	GO:0050829	Defence response to Gram‐negative bacterium	66	17	1.7e‐10
	GO:0071222	Cellular response to lipopolysaccharide	164	17	0.00012
	GO:0010466	Negative regulation of peptidase activity	163	16	0.00036
	GO:0002429	Immune response‐activating cell surface receptor signalling pathway	164	16	0.00243
	GO:1903555	Regulation of tumour necrosis factor superfamily cytokine production	137	16	0.01244
	GO:0071706	Tumour necrosis factor superfamily cytokine production	137	16	0.01244
	GO:0070665	Positive regulation of leukocyte proliferation	132	16	0.02765
	GO:0045089	Positive regulation of innate immune response	113	16	0.03224
	GO:0071356	Cellular response to tumour necrosis factor	175	15	0.00219
	GO:0002695	Negative regulation of leukocyte activation	148	15	0.01151
	GO:0002456	T cell mediated immunity	82	15	0.01605
	GO:0002705	Positive regulation of leukocyte mediated immunity	113	15	0.01837
	GO:0032680	Regulation of tumour necrosis factor production	133	15	0.03262
	GO:0032640	Tumour necrosis factor production	133	15	0.03262
	GO:0050866	Negative regulation of cell activation	165	15	0.04048
*Drosophila*	GO:0000819	Sister chromatid segregation	140	11	0.02927
	GO:0070192	Chromosome organisation involved in meiotic cell cycle	54	9	0.00849
	GO:0007131	Reciprocal meiotic recombination	37	7	0.00066
	GO:0007143	Female meiotic nuclear division	54	6	0.02270
	GO:0035967	Cellular response to topologically incorrect protein	44	5	0.03334
	GO:0035966	Response to topologically incorrect protein	47	5	0.04266
	GO:0007141	Male meiosis I	13	4	0.00150
	GO:0140543	Positive regulation of piRNA transcription	3	3	6.9e‐05
	GO:0010526	Retrotransposon silencing	8	3	0.00331
	GO:0007130	Synaptonemal complex assembly	10	3	0.00666
	GO:0030719	P granule organisation	11	3	0.00888
	GO:0071218	Cellular response to misfolded protein	12	3	0.01149
	GO:0051788	Response to misfolded protein	12	3	0.01149
	GO:0007135	Meiosis II	15	3	0.02169
	GO:0034508	Centromere complex assembly	19	3	0.04094

*Note:* The extended version of the table, which also includes the expected number of annotated genes per GO term and all the other enriched GO terms, can be accessed in Table S7.

### 3.4 Selection Analyses

All the DSFGs that were analysed for the AASD were also tested for positive or relaxed selection, to check whether highly divergent genes were shaped by either of the two. BUSTED returned evidence of episodic positive selection for *Sox‐C*, *Sox‐D*, *Sox‐F*, *Sox‐H*, *Fox‐C*, *Fox‐L1*, *Fox‐M*, *Fox‐O*, *Fox‐P* and *Sox‐B1/2*. RELAX returned evidence of relaxed selection (*K* < 1) for *Dmrt‐2*, *Dmrt‐3*, *Dmrt‐4/5*, *Sox‐B1/2*, *Sox‐C*, *Sox‐D*, *Sox‐F*, *Sox‐H*, *Fox‐AB*, *Fox‐C*, *Fox‐D*, *Fox‐E*, *Fox‐L2*, *Fox‐M*, *Fox‐N4*, *Fox‐O*, *Fox‐OG13/NA*, *Fox‐OG16/N/A*, *Fox‐P*, *Fox‐Q2a*, *Fox‐Q2b* and *Fox‐Q2c*, while evidence of intensified selection (*K* > 1) for *Fox‐A*, *Fox‐B*, *Fox‐J1*, *Fox‐J2/3*, *Fox‐L1*, *Fox‐N3*, *Sox‐B1/2* and *Sox‐E* (Table S8). Therefore, among the highly divergent DSFGs (i.e., *Dmrt‐1L*, *Sox‐H* and *Sox‐F*), only *Sox‐H* and *Sox‐F* were found to have been shaped by either positive selection or relaxed selection.

### 3.5 Dmrt, Sox and Fox Genes, and Amino Acid Sequence Divergence in the Test Datasets

**FIGURE 4:**
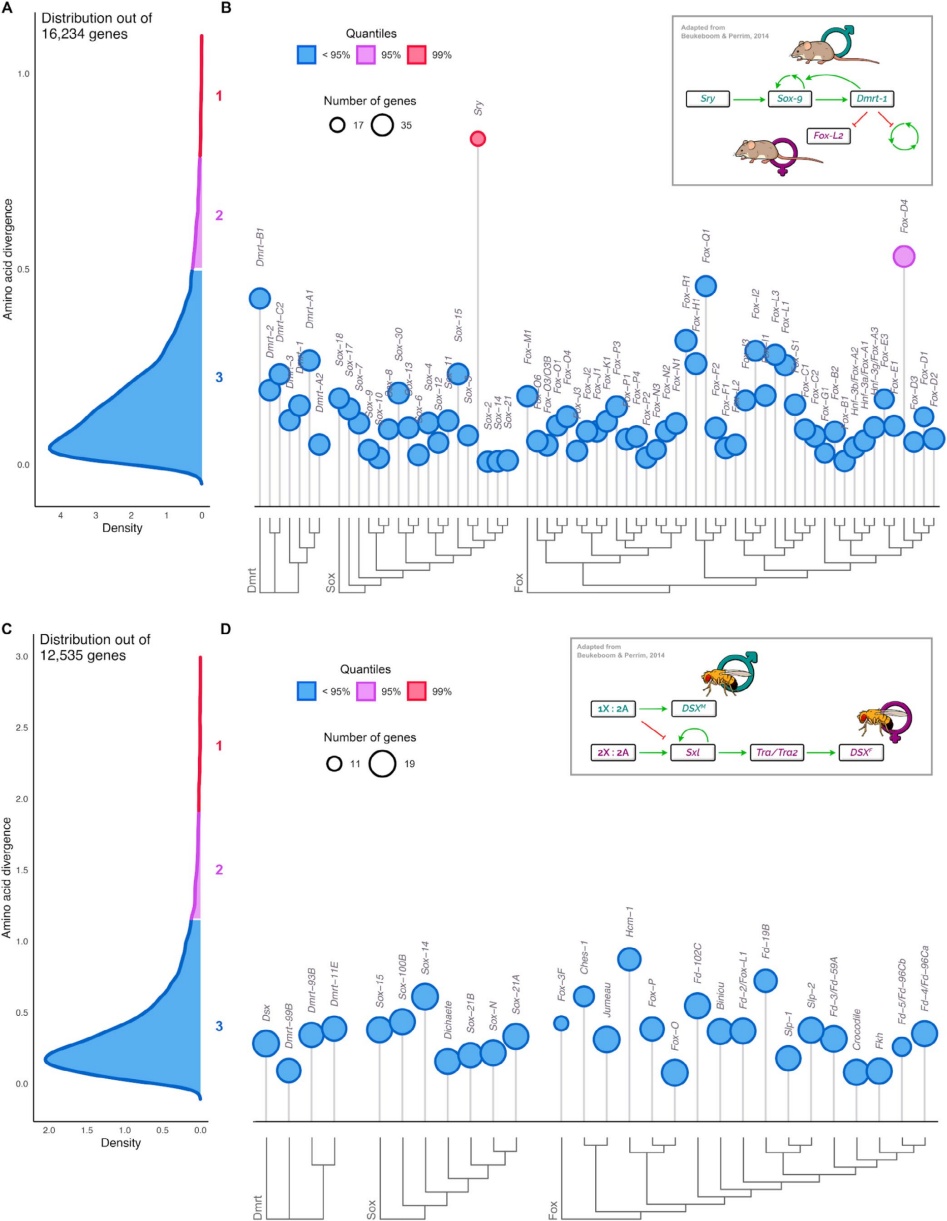
Distribution of amino acid divergence (AASD) of single‐copy orthogroups in mammals (A) and fruit flies (C), including Dmrt, Sox and Fox genes (DSFGs; B–D). The distributions of AASD in mammals and fruit flies have been computed on the median values of pairwise distances of over 16 k and 12 k SCOs, respectively. Genes have been divided according to their median AASD value into three different groups, which are indicated by different colours and increasing numbers (Groups 1, 2 and 3). Genes from Group 1 and Group 2 are collectively named ‘highly divergent genes’. Circle heights of DSFGs show the median value of their AASD, while the size indicates the number of represented species. DSFG trees are shown on the bottom; note that the Dmrt gene trees are unrooted, as at the moment no current outgroup is known for the gene family (full trees can be found in Figures S6–, S8 for mammals and in Figures S9–, S11 for fruit flies). In *Drosophila*, *Sxl* and *tra*, both involved in the SD pathway (inset) do not belong to the group of highly divergent genes (mean amino acid divergence of 0.092 and 0.978, respectively; that is, within the boundaries of Group 3). Insets: Scheme of the sex‐determination molecular pathways in *Mus musculus* (top) and in *Drosophila melanogaster (bottom)* , showing the main genes involved (adapted from Beukeboom and Perrin 2014). Green arrows indicate transcription activations, red arrows indicate transcription suppressions. A, autosomal chromosomes; *DSX* ^M/F^, *DSX* splicing variants present in males or females, respectively; X, sex chromosomes. Trees were plotted using the R package ‘ggtree’ (Yu, Smith, et al. 2017).

Most of the already‐recognised DSFG orthology groups in mammals and fruit flies have been identified. In mammals, we retrieved 7 Dmrt orthology groups, 20 Sox orthology groups, and 42 Fox orthology groups (Figures S5A, [Link], [Link]; Table S9). Of these, just *Sox‐5* was not included in the AASD analysis, as it did not meet the 50% species occupancy threshold. OrthoFinder analysed ~650 M genes, and the number of SCOs used in the AASD analysis is > 16 k (Figure 4A). From the distribution of median AASD, 163 genes were assigned to Group 1, 649 to Group 2, and 15,355 to Group 3. Most of the DSFGs (66/68) fell in Group 3 (Figure 4B), while *Sry* and *Fox‐D4* showed higher divergences and have been accordingly placed in Group 1 and Group 2, respectively. Genes from Group 1 and Group 2 show a strong enrichment in immune‐related functions (such as innate and adaptive immune response, defence response to bacteria and viruses, lymphocyte metabolism, etc.), but also in reproductive processes (such as spermatogenesis; Tables 1 and S7). Concerning *Drosophila*, we retrieved 4 Dmrt orthology groups, 7 Sox orthology groups, and 17 Fox orthology groups (Figures S5B, S10–, S11, Table S10). OrthoFinder analysed ~240 M, and the distribution of median AASD was built after > 12 k SCOs (Figure 4C). 126 genes were assigned to Group 1, 501 to Group 2, and 11,880 to Group 3. All the DSFGs have been used in the AASD analysis, but none of them have been placed in Group 1 or Group 2; that is, all the DSFGs in *Drosophila* have an AASD comparable to the median level of the genome (Figure 4D). Genes of Group 1 and Group 2 show a GO‐term enrichment in meiotic processes, such as chromosome/chromatid organisation and retrotransposon silencing (Tables 1 and S7).

## 4 Discussion

### 4.1 A New Manually‐Curated and Phylogenetic‐Based Reference Dataset of Dmrt, Sox, and Fox Genes in Bivalves

The annotation and characterisation process of a gene family may harbour many overlooked challenges in a certain clade of organisms (Vizueta et al. 2020). For example, the presence of highly conserved catalytic domains may hamper the correct identification of the components of a gene family because of insufficient phylogenetic signal, as is the case for Hox and ParaHox genes and their homeobox motif (Baldwin‐Brown et al. 2018; Nicolini, Martelossi, et al. 2023). Conversely, the components of dynamic gene families characterised by abrupt and sequential duplication events may be difficult to sort into separate groups. As a matter of fact, varying levels of sequence heterogeneity and gene copy numbers make the inference of orthologous groups hard, as for certain clans of the P450 family (Dermauw et al. 2020). Regardless of the causes, having a solid and wide phylogenetic context in which to study gene duplications/losses and orthology relationships is crucial to overcome these difficulties. In the same way, manual curation and visual inspection of multiple sequence alignments, phylogenetic trees and gene structures (in terms of domain composition, start and stop codons and other feature representations) are helpful, despite being time‐demanding and possibly less reproducible. In this study, we characterised the full complement of DSFGs in the vast class of bivalves by leveraging sequence domain annotation, phylogenetics and manual curation of the dataset. Our aim was to obtain the most reliable gene complements possible, combined with a vast taxonomic dataset, a solid phylogenetic inference, an openly available dataset of gene sequences and a reproducible pipeline for the annotation of gene identity. By doing so, we wanted to provide a reliable resource for future studies of DSFGs, either focused on bivalves or on Metazoa in general.

Concerning the Dmrt gene family, we identified orthologs of the vertebrate *Dmrt‐2*, *Dmrt‐3* and *Dmrt‐4/5* (*A1/A2*; Figures 2 and S1; Table S6), which are also expected to have been present in the Bilateria common ancestor (Mawaribuchi et al. 2019). Wang et al. (2023) found that *Dmrt‐4/5* is duplicated in *Mercenaria mercenaria* and *Cyclina sinensis* (Venerida) and in *Dreissena polymorpha* (Myida), and we confirm this result by tracing back the duplication event to the split between Palaeoheterodonta (here represented by Unionida) and Heterodonta (here represented by Venerida, Myida, Sphaeriida, Adapedonta, Cardiida and Lucinida; Figure 2). Furthermore, we confirm *Dmrt‐1L* to be present in many bivalve species (mainly belonging to the Ostreida, Pectinida, Mytilida and Unionida orders; Figure 2; Table S6), as well as in gastropods and *Octopus*. However, our phylogenetic analysis did not retrieve any unambiguous orthology relationship between *Dmrt‐1L* and either vertebrate *Dmrt‐1* or *Drosophila dsx* genes, as instead it was proposed in previous works (Li et al. 2018; Evensen et al. 2022). As a matter of fact, the amino acid sequence of the *Dmrt‐1L* DM domain does not recall that of any other Dmrt gene. Furthermore, it must be considered that various phylogenetic analyses have recovered both *Dmrt‐1* and *dsx* genes to be restricted to vertebrates and arthropods, respectively (Wexler et al. 2014; Mawaribuchi et al. 2019; Panara et al. 2019); that is, they do not have any direct ortholog outside their relative clades. Thus, if *Dmrt‐1L*, *dsx* and *Dmrt‐1* were true orthologs, their origin would need to be placed at least in the Bilateria common ancestor, which seems, however, to not be the case. All considered, we thus confirm that *Dmrt‐1L* is not orthologous to *Dmrt‐1* and *dsx* and is rather a mollusc‐specific gene (Evensen et al. 2022). The monophyly of the *Dmrt‐1L* group is not supported by the phylogenetic tree inferred with Dmrt genes from molluscs and the reference species (Figure S1); however, it is recovered when analysing just genes from mollusc species (Figure S12). In this regard, we speculate that in our analysis, the difficulty in obtaining the monophyly of *Dmrt‐1L* genes may have arisen primarily because of the many *C. elegans* ‐restricted genes (Table S3), which are placed among bivalve genes (Figure S1), but also because of the high AASD of *Dmrt‐1L* genes (see High Amino Acid Sequence Divergence Identifies Putative Sex‐Determining Genes), which hampers a straightforward phylogenetic reconstruction. Furthermore, our broad‐context analysis allowed us to identify some cases of incorrect gene identification in bivalves, which have arisen because of erroneous or ambiguous annotations in previous works, as a result of limited datasets or analyses. For example, (i) the scallop‐specific cluster of Dmrt genes retrieved by Wang et al. (2023) rather belongs to the *Dmrt‐1L* group, and (ii) the classification of Dmrt genes in *Crassostrea* species provided by Zeng et al. (2024) needs to be revised as follows: *Dmrt‐1* genes are *Dmrt‐4/5*; *Dmrt‐2* genes are *Dmrt‐3*; *Dmrt‐3* genes are *Dmrt‐1L* (Table S6); hence, *Crassostrea* species do not have *Dmrt‐2* genes.

For what concerns the Sox gene family, bivalves (or molluscs) do not show any major clade‐restricted gene, as only the five Bilateria‐specific Sox groups (*Sox‐B1/2*, *Sox‐C*, *Sox‐D*, *Sox‐E* and *Sox‐F*) and *Sox‐H* have been identified (Figures 2 and S2; Table S6), in accordance with previous findings (Yu, Zhang, et al. 2017; Evensen et al. 2022; Wang and Nie 2024; Zhang et al. 2025). *Sox‐B1/2* is clearly made up of two subgroups (i.e., *Sox‐B1* and *Sox‐B2*), as expected, but their respective identity could not be unambiguously established, as *Sox‐B1/2* genes of reference species do not form separate clusters (Figure S2), even when inferring the phylogenetic tree only of components of the *Sox‐B1/2* group from molluscs and reference species (Figure S13).

Compared to Dmrt and Sox genes, the Fox gene family appears as the most dynamic in terms of gene presence/absence, as already shown by other works (Wu et al. 2020; Schomburg et al. 2022; Seudre et al. 2022). Our phylogenetic analysis successfully recovered Group I and Group II of Fox genes (Larroux et al. 2008), which include the four Fox genes that were present in the Bilateria common ancestor (*Fox‐C*, *Fox‐F*, *Fox‐L1* and *Fox‐Q1*; Figures 2 and S3; Table S6; Shimeld et al. 2010). To our knowledge, this is the first broad taxonomic identification and classification of Fox genes in bivalves, as up to now they have been systematically characterised only in *C. gigas* (Yang et al. 2014), *Patinopecten yessoensis* (Wu et al. 2020), and *Ruditapes philippinarum* (Liu et al. 2024). Firstly, our analysis confirms the absence in molluscs of *Fox‐I*, *Fox‐Q1*, *Fox‐R*, *Fox‐S* (Figure S3), which are in fact thought to have emerged with the diversification of deuterostomes or vertebrates (Yang et al. 2014; Wu et al. 2020; Schomburg et al. 2022; Seudre et al. 2022). Furthermore, we have found many Fox groups that appeared as mollusc‐specific and/or still unnamed at a first analysis. However, a more in‐depth investigation revealed a different scenario. *Fox‐OG2/NA* appears close to the human *Fox‐M* gene in the phylogenetic tree, but they do not form a monophyletic group (Figure S3). However, by comparing *Fox‐OG2/NA* sequences and the phylogenetic tree with those analysed by Yang et al. (2014), Wu et al. (2020), Schomburg et al. (2022), Seudre et al. (2022), it is clear that this group of Fox genes is indeed *Fox‐M* (Table S6). However, our analysis has failed to retrieve a monophyletic relationship among bivalve and human *Fox‐M* genes, even when inferring a tree with just *Fox‐J2*, *Fox‐M*, *Fox‐O* and *Fox‐P* complements (Figure S14), which belong to the same Fox group. Regarding the *Fox‐OG39/NA* group, it does not have any homologue in reference species (Figure S3) but is found to belong to the *Fox‐AB* group by sequence comparison with previous works (Yang et al. 2014; Wu et al. 2020; Seudre et al. 2022). *Fox‐AB* was formerly described only in the sea urchin *Strongylocentrotus purpuratus* and the lancelet *Branchiostoma floridae* (Tu et al. 2006; Yu et al. 2008), but was later identified also in several Spiralia lineages, including molluscs (e.g., Yang et al. 2014; Wu et al. 2020; Seudre et al. 2022). A similar situation concerns *Fox‐OG15/NA* and *Fox‐OG28/NA*, which again could not be named based on orthology relationships with the reference species genes (Figure S3) but actually represent two lineage‐specific expansions of the *Fox‐Q2* group (named *Fox‐Q2b* and *Fox‐Q2c*; Table S6), as already appointed in previous studies (Yang et al. 2014; Wu et al. 2020). This observation fits within the wider context of the *Fox‐Q2* group expansion in Bilateria and, particularly, in Spiralia, that led to remarkable differences in their gene copy numbers across various clades (Seudre et al. 2022). Two additional Fox genes have been previously identified in bivalves and were named *Fox‐Y* and *Fox‐Z* (Yang et al. 2014; Wu et al. 2020). In our analysis, these Fox genes were identified as *Fox‐OG13/NA* and *Fox‐OG16/NA* (Table S6), after sequence comparison with Fox genes from *C. gigas* and *P. yessoensis* . On one hand, *Fox‐Y* was first identified in *S. purpuratus* (Tu et al. 2006) and only recently in a few bivalve species (Yang et al. 2014; Wu et al. 2020). However, when analysing bivalve and *S. purpuratus* Fox genes, we failed in retrieving such a clear orthology relationship, as *S. purpuratus Fox‐Y* does not fall within the phylogenetic range of bivalve *Fox‐OG13/NA*, which contains the supposed *Fox‐Y* orthologs (Figure S15). Also, the forkhead domains of *Fox‐OG13/NA* genes were annotated as ‘forkhead domain P’ (Table S6). On the other hand, *Fox‐Z* was first identified in bivalves and in several other protostomes, thanks to a phylogenetic work including the brachiopod *Lingula unguis*, the annelid *Capitella teleta*, the scorpion *Centruroides sculpturatus*, and the centipede *Strigamia maritima* (Wu et al. 2020). However, later works have not recovered this Fox gene, even when analysing annelids (Seudre et al. 2022) and panarthropods (Schomburg et al. 2022) in a more focused effort. In this case, the forkhead domains were annotated as either a generic ‘forkhead domain’ or a ‘forkhead domain Q2’ (Table S6). All considered, we argue that bivalves possess two additional Fox groups (here *Fox‐OG13/NA* and *Fox‐OG16/NA*; Figures 2 and S3; Table S6) which are shared with other mollusc species, as revealed also by other authors. However, given the discordant results of the phylogenetic hypothesis and domain annotation, we think that a more thorough investigation of their orthology relationships with Fox genes from other Metazoa is needed, and thus we chose to not employ their former names, *Fox‐Y* and *Fox‐Z*.

Besides the DSFG groups discussed so far, it must be also considered that many orphan genes have been identified (Figure S1–, S3; Table S6). For example, Wu et al. (2020) identified a duplication event of *Fox‐H* genes in *C. gigas* , which has been recovered also in our analysis for the entire Ostreida clade (*Fox‐OG36/NA*; Figure S3). Similarly, a gene orthology group putatively specific to Pteriomorphia has been identified among Sox genes (*Sox‐OG1/NA*). Of course, these genes deserve as much attention as their widely distributed paralogs, as they may constitute true group‐specific expansions and may play fundamental roles in some biological processes. However, they have not been discussed here or included in Figure 2 for clarity purposes, but their sequences are available on GitHub and Dryad (see the Data Availability Statement). In the same way, it must be noted that cases of missing genes can be interpreted either as true biological absence or as technical artefacts. The former case is difficult to assess in a reliable way, unless, for example, an entire clade is affected (as is the case for *Dmrt‐1L*, which has been lost in all Heterodonta investigated here [see Results and Figure 2]). To confirm that a certain gene is absent from one or more species, a much more integrated investigation is needed. This is particularly true when talking about transcriptomes, whose completeness in terms of gene content can be strongly affected by the sequencing condition (e.g., a gene can be missing from a transcriptome just because it was not transcribed in the sampled material). In these cases, it is fairly impossible to declare that a gene is absent without at least a supporting genome.

Overall, our analysis clearly shows the importance of adopting a wide‐angle approach when characterising the members of a gene family, especially for large ones such as the Fox genes (Schomburg et al. 2022). As a matter of fact, the presence of duplication events and orphan genes needs to be addressed with a broad taxonomic dataset in order to account for possible mis‐annotations, gene phylogenetic misplacements and sequence heterogeneity. Additionally, many reference species need to be included for the gene identification process to consider distantly related genes and obtain a solid annotation. Our gene annotation pipeline resulted to be very solid, even with non‐model organisms and sub‐optimal genomic and transcriptomic resources, such as those of bivalves. As a matter of fact, by running the same pipeline on two additional datasets composed of mammal and fruit fly genomes, we were able to obtain high‐quality orthology groups in accordance with previous knowledge on the clades (Figures S5–, S11; Tables S9–S10), with little or no need for manual curation. Furthermore, the present work represents the first broad analysis of DSFGs in both mammals and fruit flies, as so far attention has been mainly dedicated to single well‐studied organisms or little clades (e.g., Jackson et al. 2010).

### 4.2 High Amino Acid Sequence Divergence Identifies Putative Sex‐Determining Genes

Sex‐biased genes tend to evolve more rapidly than unbiased genes at the level of their protein sequences. Accelerated rates have been observed in both male‐biased genes (reviewed in Parsch and Ellegren 2013; Grath and Parsch 2016) and female‐biased genes (e.g., Papa et al. 2017; Ghiselli et al. 2018), but also in SRGs and SDGs (O'Neil and Belote 1992; Whitfield et al. 1993; de Bono and Hodgkin 1996). For example, it has been shown that *Dm‐W*, *Dmy* and *Sry* (which are SDGs in the African clawed frog *X. laevis* , in the medaka fish *O. latipes* , and in eutherians, respectively) all have higher substitution rates than their paralogues (*Dmrt‐1* for *Dm‐W* and *Dmy*, *Sox‐3* for *Sry*), particularly when considering their DNA‐binding domains (Mawaribuchi et al. 2012). Similarly, both a burst of positive selection and a relaxation of purifying selection have been detected in *Drosophila Sxl* in correspondence with its recruitment at the top of the sex‐determining cascade. The same signs of relaxed purifying selection have been found in the downstream targets of *Sxl*, that is, *transformer* (*tra*) and *dsx*, despite no evidence of positive selection being detected (Mullon et al. 2012).

Considering these shared features of SRGs and SDGs, we decided to look for signs of accelerated sequence evolution in DSFGs of bivalves to evaluate if any of them could be a priori associated with SD by employing the tools of molecular evolution. However, we analysed patterns of sequence evolution not only among putative SRGs and their close paralogs but also considering the genomic context in which these genes evolve. Our aim was to check whether SRGs show higher rates of sequence evolution when compared to other genes not involved in SD and not belonging to the same gene family. To do so, we obtained the AASD median values of more than 11 k SCOs from bivalve genomes (Figure 3A), in order to build a statistical distribution to be used as a reference: if SRGs/SDGs (in this case, DSFGs) truly evolve faster than other genes, we may expect them to fall within the 5% (or even 1%) upper quantile of the distribution (Figure 3B), i.e., within highly divergent genes (Group 1 and Group 2 genes of the distribution). We chose to use the AASD as a metric of sequence evolution (instead of the tip‐to‐tip distances of phylogenetic trees, which account for more comprehensive evolutionary models) to save computational time. As a matter of fact, the AASD median values proved to be a good approximation of the tip‐to‐tip median distances in 200 randomly selected genes (Figure 3C; *R* = 0.84, *p* < 2.2E−6).

Among DSFGs, three fell within the 5% upper quantile, namely *Dmrt‐1L*, *Sox‐H* and *Sox‐F*. Interestingly, on the basis of DGE analyses, *Dmrt‐1L* and *Sox‐H* have already been proposed to be involved in the male SD pathway of *C. gigas* (inset in Figure 3B; Zhang et al. 2014), while *Sox‐H* has also been indicated as a candidate gene involved in the development of male germ cells in the Manila clam *Ruditapes philippinarum* (Ghiselli et al. 2012). Specifically, *Sox‐H* would play a major role in *C. gigas* SD by interacting with *Dmrt‐1L* and determining the onset of male phenotype development; at the same time, both *Sox‐H* and *Dmrt‐1L* would inhibit *Fox‐L2*, which instead is necessary to start female phenotype development. *Dmrt‐1L* and *Sox‐H* have been appointed several other times to be involved in male gonad development and differentiation through DGE (e.g., Teaniniuraitemoana et al. 2014; Capt et al. 2018; Afonso et al. 2019; Zhang et al. 2025), ISH (e.g., Naimi et al. 2009; Li et al. 2018; Liang et al. 2019; Yue et al. 2021) and RNA interference (Liang et al. 2019; Sun et al. 2022). Therefore, the high AASD of *Dmrt‐1L* and *Sox‐H* is coherent with previous works, strengthening their role as putative SRGs.

The relationship between high gene AASD and the involvement in SD is particularly strengthened when looking at the patterns of AASD in the test datasets, which corroborates the solidity of our analysis: (i) in the mammal dataset—which represents a strictly genetic SD system with a master and rapidly evolving SDG—one of the genes from the 5% upper quantile of the distribution is *Sry* (Figure 4A,B), the male sex‐determining gene in eutherians (inset in Figure 4B); (ii) in the fruit fly dataset—which represents a chromosomic SD system and has no differences in the rates of sequence evolution among SRGs—none of the DSFG exhibit significantly higher AASD (Figure 4C,D), including the downstream effector *dsx* (inset in Figure 4D). Also, *Sxl* and *tra*, both involved in the SD pathway of *Drosophila* (inset in Figure 4D), do not belong to the group of highly divergent genes, as they have a mean amino acid divergence of about 0.09 and 0.9, respectively (Group 3; Figure 4D). Therefore, it can be argued that both *Dmrt‐1L* and *Sox‐H* may not only be SRGs, but may participate in bivalve SD as primary SDGs, which is reflected in their high AASD, as it is observed for *Sry* in mammals. As a matter of fact, if they were involved in SD just as intermediate actors of the signalling cascade, we should not have observed a high AASD, as *Drosophila Sxl*, *tra* and *dsx* seem to suggest. Overall, these patterns of molecular evolution concerning SRGs and SDGs are also supported by the way SD regulatory networks evolve. It has been proposed that the sex‐determining cascades tend to arise and be established with a bottom‐up mechanism (Wilkins 1995; Mullon et al. 2012; Beukeboom and Perrin 2014; Capel 2017). This means that the regulatory relationships among genes at the bottom of the cascade are settled prior to the regulatory relationships among genes at the top and, consequently, upstream regulators are progressively recruited to fine‐tune diverse SD signals. These evolutionary patterns eventually produce gene regulatory networks in which the divergence of the upstream triggers is higher than that of downstream effectors, in terms of both identity and sequence composition (Beukeboom and Perrin 2014). This mechanism has been proposed for *Drosophila* species (Mullon et al. 2012), *C. elegans* (Stothard and Pilgrim 2003) and vertebrates, although in the latter case it has been questioned several times (reviewed in Capel 2017).

Two main objections can be moved against our approach: (1) the distribution of AASD is not appropriate for this kind of inference, as it does not represent the true gene evolutionary (or substitution) rates (which instead are those usually utilised when dealing with SRGs and SDGs); (2) the three datasets are not comparable one to another, as they take into consideration very different animal groups, with different taxonomic rankings and different divergence times (thus, the patterns of AASD may be the products of other confounding factors not directly related to SD). Concerning the first objection, we are aware that the AASD does not represent the evolutionary rate itself, but rather its product. However, the two features are tightly linked, as over the long term highly divergent proteins tend to be produced by genes with high evolutionary (or substitution) rates (Echave et al. 2016). By performing a GO‐term enrichment, it emerged that highly divergent genes of the mammal dataset are mainly involved in the immune response and male spermatogenesis (Tables 1 and S7), which are two processes notoriously connected with rapid sequence evolution (i.e., higher evolutionary rates; Swanson and Vacquier 2002; Murat et al. 2023; Vinkler et al. 2023). Similarly, highly divergent genes from the fruit fly dataset show an enrichment for GO terms associated with meiotic‐related functions (such as the formation of the synaptonemal complex by the products of *c(2)M*, *c(3)G*, *corona* and *corolla* genes; Tables 1 and S7), which again are known to evolve rapidly (Hemmer and Blumenstiel 2016). Similarly, after having manually inspected fruit fly genes, we noticed that some genes encoding seminal fluid proteins (e.g., *Acp53C14c*, *Sfp24F*, *Sfp35C*, *Sfp53D* and *Sfp84E*; sequences compared with those from Wigby et al. 2020) are included among highly divergent genes, as already noticed by previous works (e.g., Thayer et al. 2024). In other words, the test datasets—which include well studied and characterised model systems—allow us to directly link the high AASD (as computed in this work) with high rates of sequence evolution (as found in previous works). This consideration can thus be extended also to the bivalve dataset: highly divergent genes in terms of AASD, which include some DSFGs and show an enrichment for GO terms associated with macromolecule metabolism and morphological development (Tables 1 and S7), are also genes with accelerated substitution rates (Ghiselli et al. 2018; Iannello et al. 2023).

Concerning the second objection, we chose two test datasets with different characteristics, as we wanted to check the extent of our hypothesis, that is, molecular evolution can be used to look for putative primary SDGs in taxonomic‐wide analyses. The difference in divergence times and taxonomy ranks for bivalves and therians (late Cambrian [about 498 million years ago, Mya; Song et al. 2023] and early Mesozoic [166–123 Mya; Álvarez‐Carretero et al. 2022], respectively) does not seem to influence the sequence diversity of SRGs, as both *Dmrt‐1L*/*Sox‐H* for bivalves and *Sry* for mammals exhibit high AASD with respect to their own distributions, regardless of their age. *Dmrt‐1L* and *Sox‐H* (which are mollusc‐ and Bilateria‐specific, respectively) are undoubtedly older than *Sry*, which emerged in the Theria common ancestor (Foster et al. 1992), but each one of them can be considered a highly divergent gene in bivalves and mammals, respectively (i.e., genes that are included in the 5% upper quantile of bivalve and mammal AASD distributions). Conversely, the difference in divergence times and taxonomic ranks for *Drosophila* (Palaeocene/Eocene boundary [about 56 Mya; Russo et al. 2013]) may seem to be influencing the results for the dataset, resulting in a false negative. In other words, it can be argued that: (i) the genes included in the SD cascade of *Drosophila* (such as *Sxl, tra* and *dsx*; inset in Figure 4D) may have a high AASD, which, however, has not been detected by our methodological approach (e.g., this may be traced back to the young diversification age of *Drosophila* species if compared to bivalves); (ii) the species included in the analysis are congeneric, thus the sequence differentiation of SRGs may exist not at the amino acid level but at the nucleotide one. To better disentangle this issue and further discuss the fruit fly dataset, we repeated the analysis of the AASD only on species of the *Crassostrea* genus ( *C. gigas* , *Crassostrea angulata* , *Crassostrea ariakensis* and *Crassostrea virginica* ), which are much younger (Middle Cretaceous [less than 100 Mya; Qi et al. 2023]), thus comparable to *Drosophila*. Results showed that, even when analysing a smaller bivalve dataset, encompassing only 4 species of recent origin, the high AASD of *Dmrt‐1L* persists; that is, *Dmrt‐1L* is still grouped together with highly divergent genes (Figure S16). The same has not been recovered for *Sox‐H*, which fell in genes from Group 3 (the group corresponding to the 95% interval of the AASD distribution; see Materials and Methods) but still has the second highest AASD median value among DSFGs (Figure S16).

Of course, we should not expect that highly divergent genes are only those involved in SD but may also participate in other processes (as discussed earlier and shown by GO term enrichments; Tables 1 and S7). Besides the genes of interest for SD (*Dmrt‐1L*/*Sox‐H* for bivalves and *Sry* for mammals), other components of the DSFG families have been retrieved with a high AASD, despite they have never been linked directly to SD so far: *Sox‐F* in bivalves (Figure 3B) and *Fox‐D4* in mammals (Figure 4B). This implies that our approach cannot be used to unambiguously identify SDGs alone. Instead, the analysis is meant to be used to detect highly divergent genes and, subsequently, by comparison with literature and a more thorough and focused functional investigation, detect putative SDGs among them. In this view, the mammal dataset exemplifies the importance of putting the results of our pipeline (as those of any other comparative genomics analysis) into the correct evolutionary and genomic context: among DSFGs of mammals, two genes exhibit high AASD, one of which is directly related to SD (*Sry*), while the other has a function connected with neural development (*Fox‐D4*; Klein et al. 2013). Thus, the high AASD may arise either because of the involvement in the upper SD pathway or because of other life‐history traits connected with the gene, respectively. Regarding bivalves, *Dmrt‐1L* and *Sox‐H* show a sharp connection with SD as a putative primary SDG, either when considering their molecular evolutionary features or when looking at their gene expression and possible function in gonad development (Naimi et al. 2009; Teaniniuraitemoana et al. 2014; Zhang et al. 2014; Capt et al. 2018; Li et al. 2018; Afonso et al. 2019; Liang et al. 2019; Yue et al. 2021). It is difficult to further speculate on the actual involvement in SD of *Dmrt‐1L* and *Sox‐H* without any additional information on their biology, also considering that genes evolve not only at the level of their amino acid sequences but also at the levels of exon‐intron structures, indels and regulatory relationships (among others). Nonetheless, molecular evolution proves to be a valuable tool to investigate genes putatively involved in SD and to identify major targets onto which to dedicate future research effort.

Unfortunately, we were unable to assess whether the AASD of *Dmrt‐1L* and *Sox‐H* is the result of positive (diversifying) selection or relaxed selection. BUSTED and RELAX did not find any evidence in *Dmrt‐1L*, while both found significant results for *Sox‐H* (Table S8). Apparently, the analysed sequences do not bear enough signal at the nucleotide level to trace the cause of their highly diverging sequences, at least for *Sox‐H*. This is true in general for the DSFGs analysed in the present work, as many of them (*Sox‐C*, *Sox‐D*, *Fox‐C*, *Fox‐M*, *Fox‐O* and *Fox‐P*; Table S8) were found to show signals of both positive and relaxed selection. This is not surprising, as positive and relaxed selection may generate similar patterns of nucleotide substitutions in protein‐coding genes (Dapper and Wade 2020). It can be speculated that these genes are evolving under diversifying selection and relaxed purifying selection, which would promote sequence differentiation as seen in other instances (Persi et al. 2016; Zhao et al. 2024; Liu et al. 2025). However, RELAX is not designed to discriminate in shifts between positive or negative selection, as it assesses the stringency of their joint effect (Wertheim et al. 2015). Overall, the selection analyses we performed did not reveal any clear pattern among genes putatively involved in SD and with a high AASD, leaving the topic still open for future work.

## 5 Conclusions

Genes functioning in reproductive processes, and particularly SDGs, are often among the most variable in animal genomes, in terms of both sequence composition and regulatory interactions (Swanson and Vacquier 2002; Bachtrog et al. 2014; Dapper and Wade 2020). Such high evolutionary rates may be traced back both to adaptive evolution (either as natural or sexual selection) or to non‐adaptive processes (Vicoso and Charlesworth 2006; Meisel and Connallon 2013; Parsch and Ellegren 2013; Grath and Parsch 2016; Dapper and Wade 2020) and often result in striking differences in reproductive and sexual systems even among closely related species. In the present work, we took advantage of this characteristic to identify SDGs among the DSFG families in bivalves. By comprehensively analysing the phylogenetic history and AASD in a broad taxonomic dataset, we appointed *Dmrt‐1L* and *Sox‐H* as putative SDGs, thus confirming results in previous works that found them to be transcribed in a male‐biased manner and/or strongly involved in male‐gonad formation (Naimi et al. 2009; Teaniniuraitemoana et al. 2014; Zhang et al. 2014; Capt et al. 2018; Li et al. 2018; Afonso et al. 2019; Liang et al. 2019; Yue et al. 2021). Future studies would now need to further investigate their evolutionary history. For example, considering that SRGs tend to accumulate in the genomic neighbourhood where primary SDGs are located (Capel 2017), analysing the genomic location of DSFGs in bivalve genomes may provide enlightening results. Similarly, revealing the genetic interactions of *Dmrt‐1L* and *Sox‐H* through functional and genome editing assays would undoubtedly benefit our understanding of their role in the sexual processes of bivalves. Furthermore, it must be highlighted that the diversity of DSFGs in bivalves still deserves attention in the near future, when additional omics resources are likely to become available. Only a minority of bivalves has been examined in the present work (43 out of more than 25,000 species [Bánki et al. 2025]), meaning that the diversity of SD mechanisms may increase in other undescribed systems, and the presented pattern of molecular evolution may not hold when including additional species.

## Author Contributions

F.N., A.L., F.G. and L.M. conceived the study. F.N. performed research and analysed data. L.M., A.L., F.G. and S.V.N. provided computational resources. F.N. wrote the paper. All authors edited and approved the manuscript.

## Disclosure

Benefit‐Sharing Statement: Benefits from this research accrue from the sharing of our data, codes and results on public databases as described above.

## Conflicts of Interest

The authors declare no conflicts of interest.

## Supporting information


**Figure S1:** ML phylogenetic tree of the Dmrt gene family in molluscs, including the Possvm orthology inference. For each tip, the species ID, the gene ID, the taxonomic information and the annotation as returned by the Possvm algorithm, are provided. Taxonomical information is replaced by ‘Reference’ if the sequence was used to assess orthology. Species ID can be found in Table S1. Bootstrap values are shown for each node as points colour‐coded by intervals. Major gene groups, as in Figures 2 and 3, are indicated with shaded rectangles and labels on the right of the tree.

**Figure S2:** ML phylogenetic tree of the Sox gene family in molluscs, including the Possvm orthology inference. For each tip, the species ID, the gene ID, the taxonomic information and the annotation as returned by the Possvm algorithm, are provided. Taxonomical information is replaced by ‘Reference’ if the sequence was used to assess orthology. Species ID can be found in Table S1. Bootstrap values are shown for each node as points colour‐coded by intervals. Major gene groups, as in Figures 2 and 3 are indicated with shaded rectangles and labels on the right of the tree.

**Figure S3:** ML phylogenetic tree of the Fox gene family in molluscs, including the Possvm orthology inference. For each tip, the species ID, the gene ID, the taxonomic information and the annotation as returned by the Possvm algorithm, are provided. Taxonomical information is replaced by ‘Reference’ if the sequence was used to assess orthology. Species ID can be found in Table S1. Bootstrap values are shown for each node as points colour coded by intervals. Major gene groups as in Figures 2 and 3 are indicated with shaded rectangles and labels on the right of the tree.

**Figure S4:** Distribution of amino acid sequence divergence (AASD) of single‐copy orthogroups in high‐quality bivalve genomes (BUSCO single‐copy genes > 90%) (A), including Dmrt, Sox and Fox genes (DSFGs; B), and their correlations with tip‐to‐tip distances (C), alignment lengths (D) and number of species (E). The distribution of AASD has been computed on the median values of pairwise distances of ~6000 SCOs as described for the reduced bivalve dataset (see main text and Figures 1 and 3). Genes have been divided according to their median AASD value into three different groups, which are indicated by different colours and increasing numbers (Groups 1, 2, and 3). Genes from Group 1 and Group 2 are collectively named ‘highly divergent genes’. Circle heights of DSFGs show the median value of their AASD, while the size indicates the number of represented species. DSFG trees are shown on the bottom; note that the Dmrt gene tree is unrooted, as at the moment no current outgroup is known for the gene family (full trees can be found in Figures S1–, S3). Darker points in panels C–E indicate Dmrt, Sox and Fox gene SCOs. The correlation between the amino acid distance and the tip‐to‐tip distance has been computed on 200 randomly selected orthogroups. Insets: scheme of the sex‐determination working model in *Crassostrea gigas* as hypothesised by Zhang et al. (2014), showing the main genes involved. Green arrows indicate transcription activations, and red arrows indicate transcription suppressions. Trees were plotted using the R package ‘ggtree’ (Yu, Smith, et al. 2017).

**Figure S5:** Dmrt, Sox and Fox gene (DSFG) complement in mammals and fruit flies. The presence/absence of genes in various species is indicated by filled circles. Numbers inside each circle specify genes with 2 or more copies. The shaded area highlights outgroup species, *Gallus gallus* (Aves) for mammals and *Anopheles gambiae* (Culicidae) for fruit flies. The phylogenetic tree of analysed species, as inferred from literature, is shown on the left, while major taxonomic groups are reported on the right. All species are represented by genomic data. DSFG trees are shown on the bottom; note that the Dmrt gene trees are unrooted, as at the moment no current outgroup is known for the gene family (full trees can be found in Figures S6–, S11). Full species names for both mammals and fruit flies, along with all assembly and taxonomic information, can be found in Tables S4 and S5, respectively. A.: Aves; Chirop.: Chiroptera; L.: Lagomorpha; M.: Monotremata; Me.: Metatheria; P.: Pholidota; Pe.: Perissodactyla; Prim.: Primates; Roden.: Rodentia; X.: Xenarthra; C.: Culicidae. Trees were plotted using the R package ‘ggtree’ (Yu, Smith, et al. 2017).

**Figure S6:** ML phylogenetic tree of the Dmrt gene family in mammals, including the Possvm orthology inference. For each tip, the species ID, the gene ID, the taxonomic information and the annotation as returned by the possvm algorithm, are provided. Species ID can be found in Table S4. Bootstrap values are shown for each node as points colour‐coded by intervals. Major gene groups, as in Figure S5, are indicated with shaded rectangles and labels on the right of the tree.

**Figure S7:** ML phylogenetic tree of the Sox gene family in mammals, including the Possvm orthology inference. For each tip, the species ID, the gene ID, the taxonomic information and the annotation as returned by the Possvm algorithm, are provided. Species ID can be found in Table S4. Bootstrap values are shown for each node as points colour‐coded by intervals. Major gene groups, as in Figure S5, are indicated with shaded rectangles and labels on the right of the tree.

**Figure S8:** ML phylogenetic tree of the Fox gene family in mammals, including the Possvm orthology inference. For each tip, the species ID, the gene ID, the taxonomic information and the annotation as returned by the Possvm algorithm, are provided. Species ID can be found in Table S4. Bootstrap values are shown for each node as points colour‐coded by intervals. Major gene groups, as in Figure S5, are indicated with shaded rectangles and labels on the right of the tree.

**Figure S9:** ML phylogenetic tree of the Dmrt gene family in fruit flies, including the Possvm orthology inference. For each tip, the species ID, the gene ID, the taxonomic information and the annotation as returned by the Possvm algorithm, are provided. Species ID can be found in Table S5. Bootstrap values are shown for each node as points colour‐coded by intervals. Major gene groups, as in Figure S5, are indicated with shaded rectangles and labels on the right of the tree.

**Figure S10:** ML phylogenetic tree of the Sox gene family in fruit flies, including the Possvm orthology inference. For each tip, the species ID, the gene ID, the taxonomic information and the annotation as returned by the Possvm algorithm, are provided. Species ID can be found in Table S5. Bootstrap values are shown for each node as points colour‐coded by intervals. Major gene groups, as in Figure S5, are indicated with shaded rectangles and labels on the right of the tree.

**Figure S11:** ML phylogenetic tree of the Fox gene family in fruit flies, including the Possvm orthology inference. For each tip, the species ID, the gene ID, the taxonomic information and the annotation as returned by the Possvm algorithm, are provided. Species ID can be found in Table S5. Bootstrap values are shown for each node as points colour‐coded by intervals. Major gene groups as in Figure S5 are indicated with shaded rectangles and labels on the right of the tree.

**Figure S12:** ML phylogenetic tree of the Dmrt gene family in mollusc species. For each tip, the species ID, the gene ID and the taxonomic information are provided. Species ID can be found in Table S1. The tree has been midpoint rooted. Bootstrap values are shown for each node as points colour‐coded by intervals. Major gene groups, as in Figures S2 and S3, are indicated with shaded rectangles and labels on the right of the tree.

**Figure S13:** ML phylogenetic tree of *Sox‐B1* and *Sox‐B2* genes in mollusc and reference species. For each tip, the species ID, the gene ID and the taxonomic information are provided. Taxonomical information is replaced by ‘Reference’ if the sequence was used to assess orthology. Species ID can be found in Table S1. Bootstrap values are shown for each node as points colour‐coded by intervals. Annotations of reference genes are reported next to the relative tip.

**Figure S14:** ML phylogenetic tree of *Fox‐J2*, *Fox‐M*, *Fox‐O* and *Fox‐P* genes in mollusc and reference species. For each tip, the species ID, the gene ID and the taxonomic information are provided. Taxonomical information is replaced by ‘Reference’ if the sequence was used to assess orthology. Species ID can be found in Table S1. Bootstrap values are shown for each node as points colour‐coded by intervals. Major gene groups, as in Figures S2 and S3 are indicated with shaded rectangles and labels on the right of the tree.

**Figure S15:** ML phylogenetic tree of the Fox gene family in bivalves and the sea urchin *Strongylocentrotus purpuratus* (Spur). For each tip, the species ID, the gene ID, and the taxonomic information are provided. Taxonomical information is replaced by ‘Reference’ if the sequence was used to assess orthology. Species ID can be found in Table S1. *S. purpuratus* genes are those given by Tu et al. (2006). Bootstrap values are shown for each node as points colour‐coded by intervals. Major gene groups, as in Figure S4, are indicated with shaded rectangles and labels on the right of the tree.

**Figure S16:** Distribution of amino acid sequence divergence (AASD) of single‐copy orthogroups in *Crassostrea gigas* , *Crassostrea angulata* , *Crassostrea ariakensis* and *Crassostrea virginica* (A), including DSFG (B). The distribution of AASD in *Crassostrea* has been computed on the median values of pairwise distances of over 14 k single‐copy orthogroups (SCOs). Circle heights of DSFGs show the median value of their AASD. *Dmrt‐1L* genes are indicated as ‘dmrt_disco_tree3’.

**Table S1:** Genomic and transcriptomic data of bivalves and other molluscs. For each species, the relative ID, taxonomic information, BUSCO statistics, NCBI accession number and source publication are reported.
**Table S2:** Dmrt, Sox and Fox gene (DSFG) family and domain identifiers (ID) in PANTHER and CDD, respectively. After retrieving putative DSFGs based on HMM profiles, IDs have been used to retain only reliable hits.
**Table S3:** List of DSFGs from reference species used to assess the identity of DSFGs in molluscs. NCBI accession numbers are reported in parentheses. Each row represents an orthology group.
**Table S4:** Genomic data of mammals used to retrieve DSFGs and compute amino acid sequence divergence (AASD) of single‐copy orthogroups (SCOs). For each species, the relative ID, taxonomic information, BUSCO statistics, NCBI accession number and source publication are reported.
**Table S5:** Genomic data of fruit flies of the genus *Drosophila* used to retrieve DSFGs and compute AASD of SCOs. For each species, the relative ID, taxonomic information, BUSCO statistics, NCBI accession number and source publication are reported.
**Table S6:** Complete set of DSFGs in bivalves. For each gene, the species ID (Species ID), as in Table S1, the accession number (Gene ID), the annotation and the CDD domains (including their Pssm‐ID) are indicated. Annotations are provided: (i) in the final curated form (as in Figures 2 and 3); (ii) as returned by the possvm algorithm; and (iii) as per previous works on DSFGs in bivalves.
**Table S7:** All the enriched GO terms for Group 1 and Group 2 genes of bivalves, mammals and fruit flies.
**Table S8:** BUSTED and RELAX results. For BUSTED, obtained *p*‐values are reported. For RELAX, the selection intensity parameter (*K*) and relative *p*‐values are reported. When *K* > 1, the selection strength has intensified along the selected branches. When *K* < 1, the selection has relaxed along the branches. Green cells indicate significant *p*‐values.
**Table S9:** Complete set of DSFGs in mammals. For each gene, the species ID (Species ID) as in Table S4, the accession number (Gene ID) and the Possvm‐based annotation are indicated.
**Table S10:** Complete set of DSFGs in fruit flies of the genus *Drosophila*. For each gene, the species ID (Species ID), as in Table S5, the accession number (Gene ID) and the Possvm‐based annotation are indicated.

## Data Availability

The data underlying this article and the codes used to run the analyses are available on GitHub (https://github.com/filonico/bivalvia_SRGs).Dryad reposiory Doi: https://doi.org/10.5061/dryad.0cfxpnwfj.
