# Supplementary figures and images for: Comparative Genomics of Sex‐Determination‐Related Genes Reveals Shared Evolutionary Patterns Between Bivalves and Mammals, but Not Fruit Flies
Source: Mol Ecol. 2025 Sep 21;34(20):e70103. doi: 10.1111/mec.70103 (PMC12530298; doi:10.1111/mec.70103)

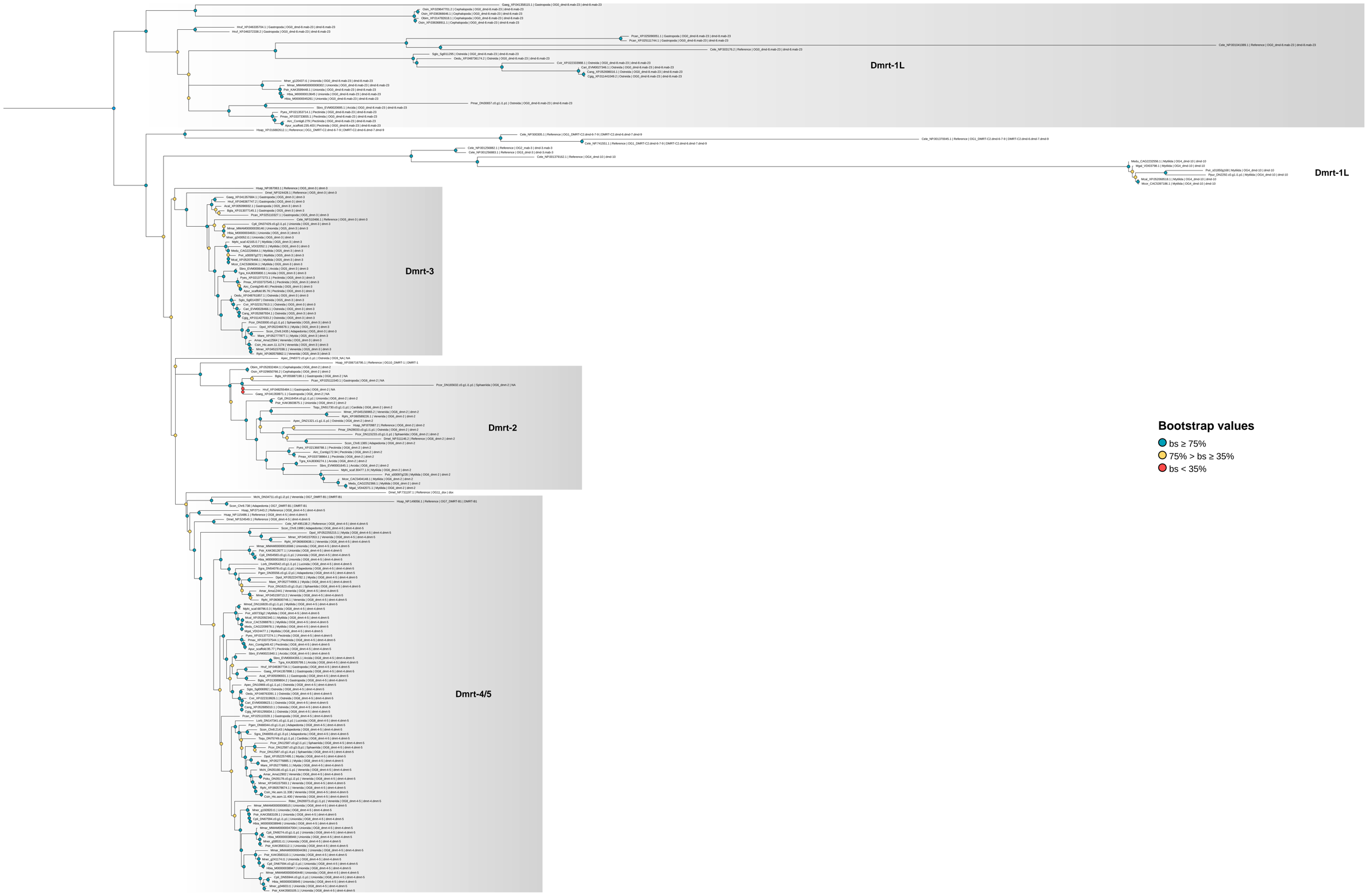

Supplement: Supplementary file 1 — Figure S1: ML phylogenetic tree of the Dmrt gene family in molluscs, including the Possvm orthology inference. For each tip, the species ID, the gene ID, the taxonomic information and the annotation as returned by the Possvm algorithm, are provided. Taxonomical information is replaced by ‘Reference’ if the sequence was used to assess orthology. Species ID can be found in Table S1. Bootstrap values are shown for each node as points colour‐coded by intervals. Major gene groups, as in Figures 2 and 3, are indicated with shaded rectangles and labels on the right of the tree. [file MEC-34-e70103-s011.pdf]

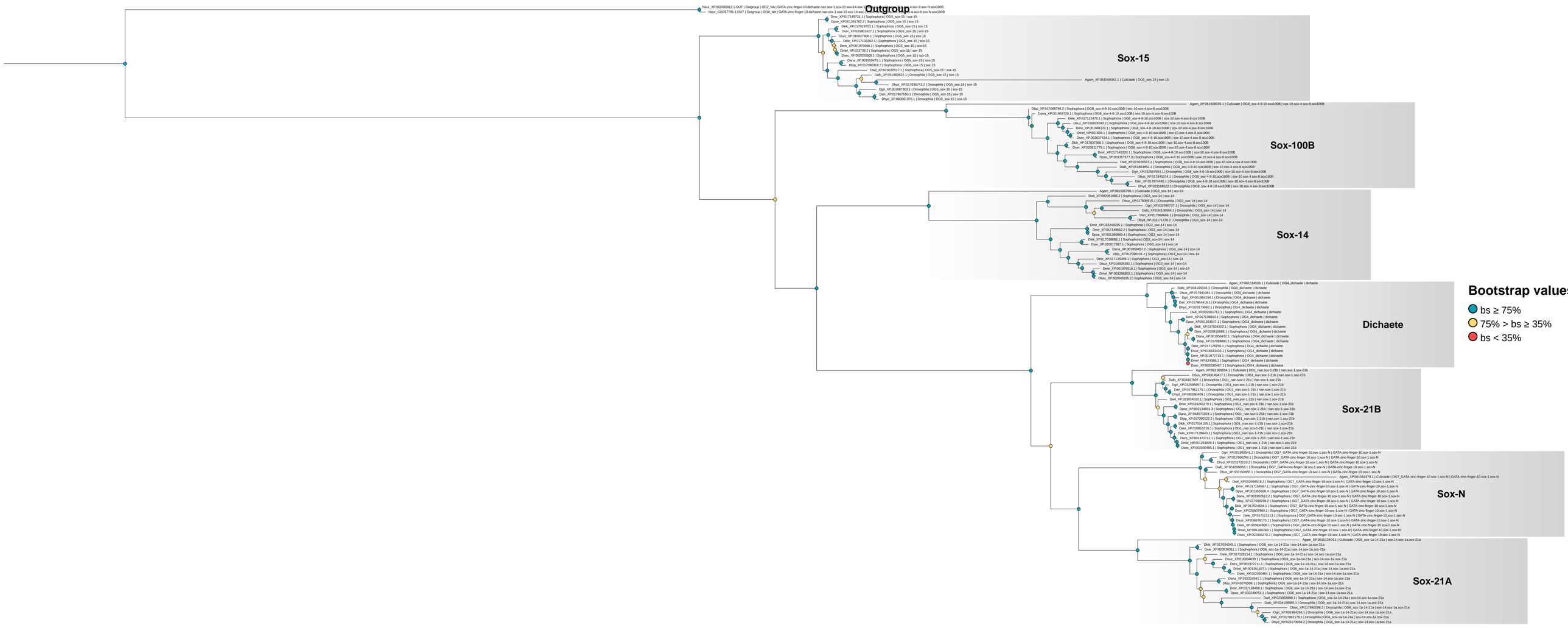

Supplement: Supplementary file 10 — Figure S10: ML phylogenetic tree of the Sox gene family in fruit flies, including the Possvm orthology inference. For each tip, the species ID, the gene ID, the taxonomic information and the annotation as returned by the Possvm algorithm, are provided. Species ID can be found in Table S5. Bootstrap values are shown for each node as points colour‐coded by intervals. Major gene groups, as in Figure S5, are indicated with shaded rectangles and labels on the right of the tree. [file MEC-34-e70103-s017.pdf]

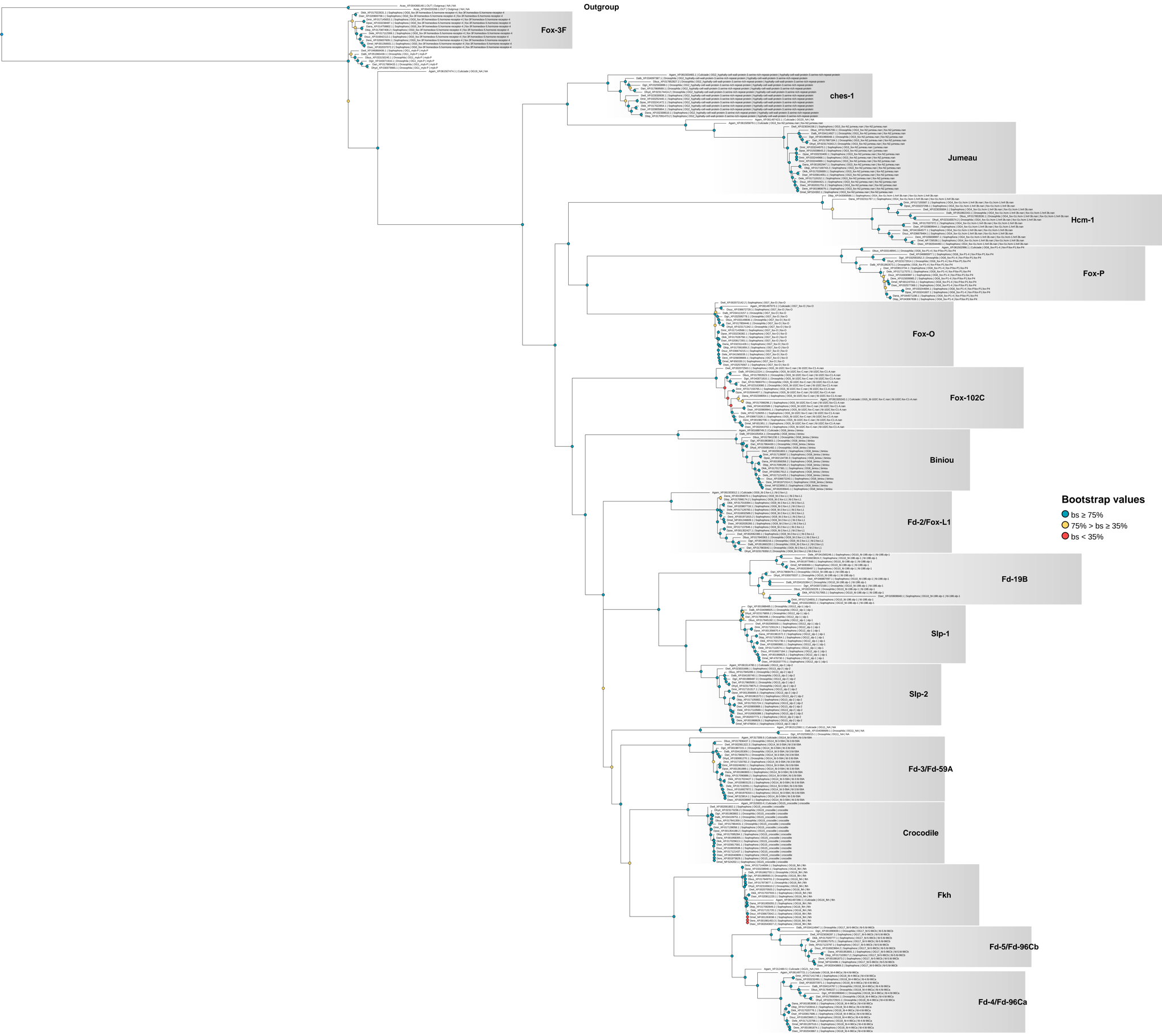

Supplement: Supplementary file 11 — Figure S11: ML phylogenetic tree of the Fox gene family in fruit flies, including the Possvm orthology inference. For each tip, the species ID, the gene ID, the taxonomic information and the annotation as returned by the Possvm algorithm, are provided. Species ID can be found in Table S5. Bootstrap values are shown for each node as points colour‐coded by intervals. Major gene groups as in Figure S5 are indicated with shaded rectangles and labels on the right of the tree. [file MEC-34-e70103-s008.pdf]

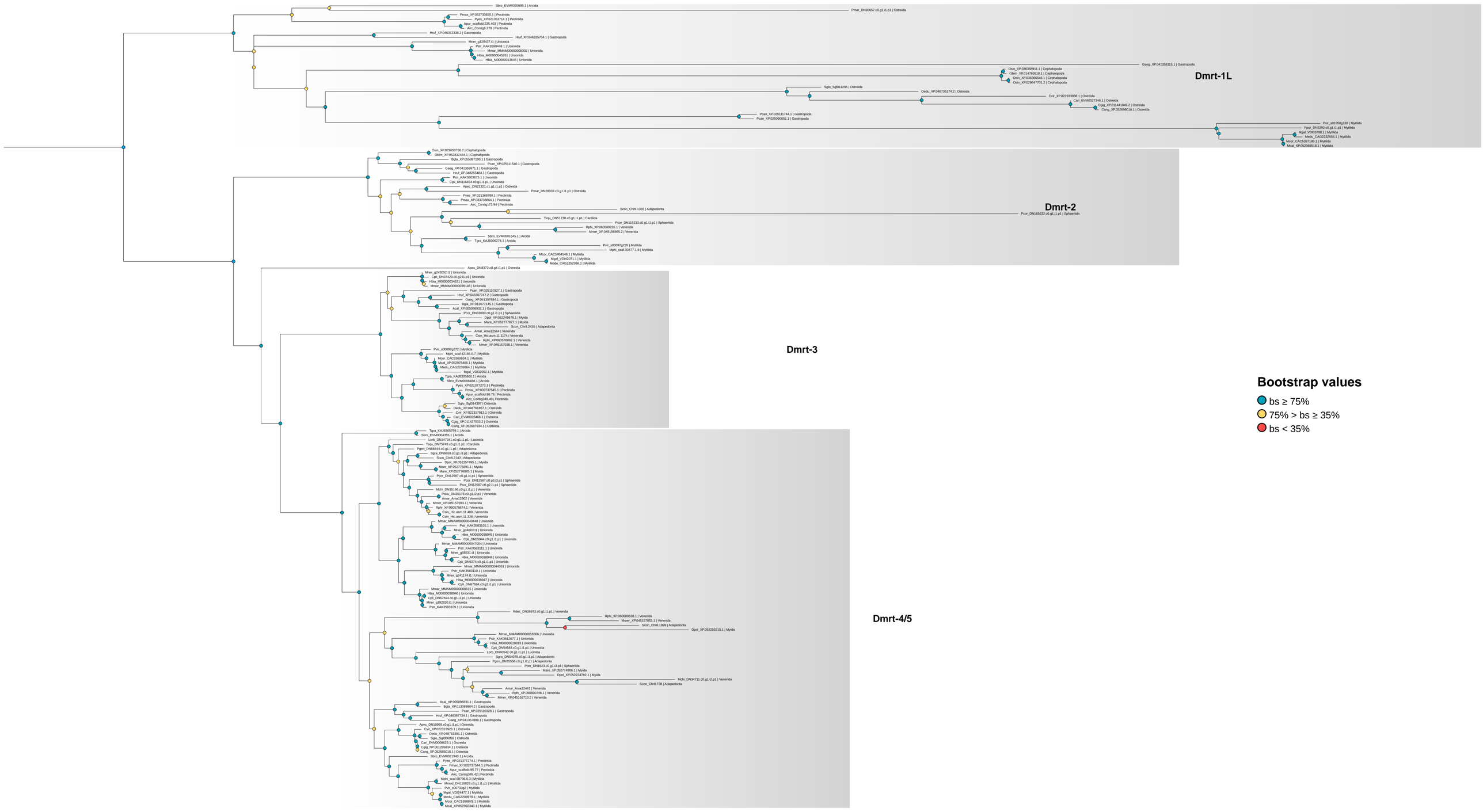

Supplement: Supplementary file 12 — Figure S12: ML phylogenetic tree of the Dmrt gene family in mollusc species. For each tip, the species ID, the gene ID and the taxonomic information are provided. Species ID can be found in Table S1. The tree has been midpoint rooted. Bootstrap values are shown for each node as points colour‐coded by intervals. Major gene groups, as in Figures S2 and S3, are indicated with shaded rectangles and labels on the right of the tree. [file MEC-34-e70103-s014.pdf]

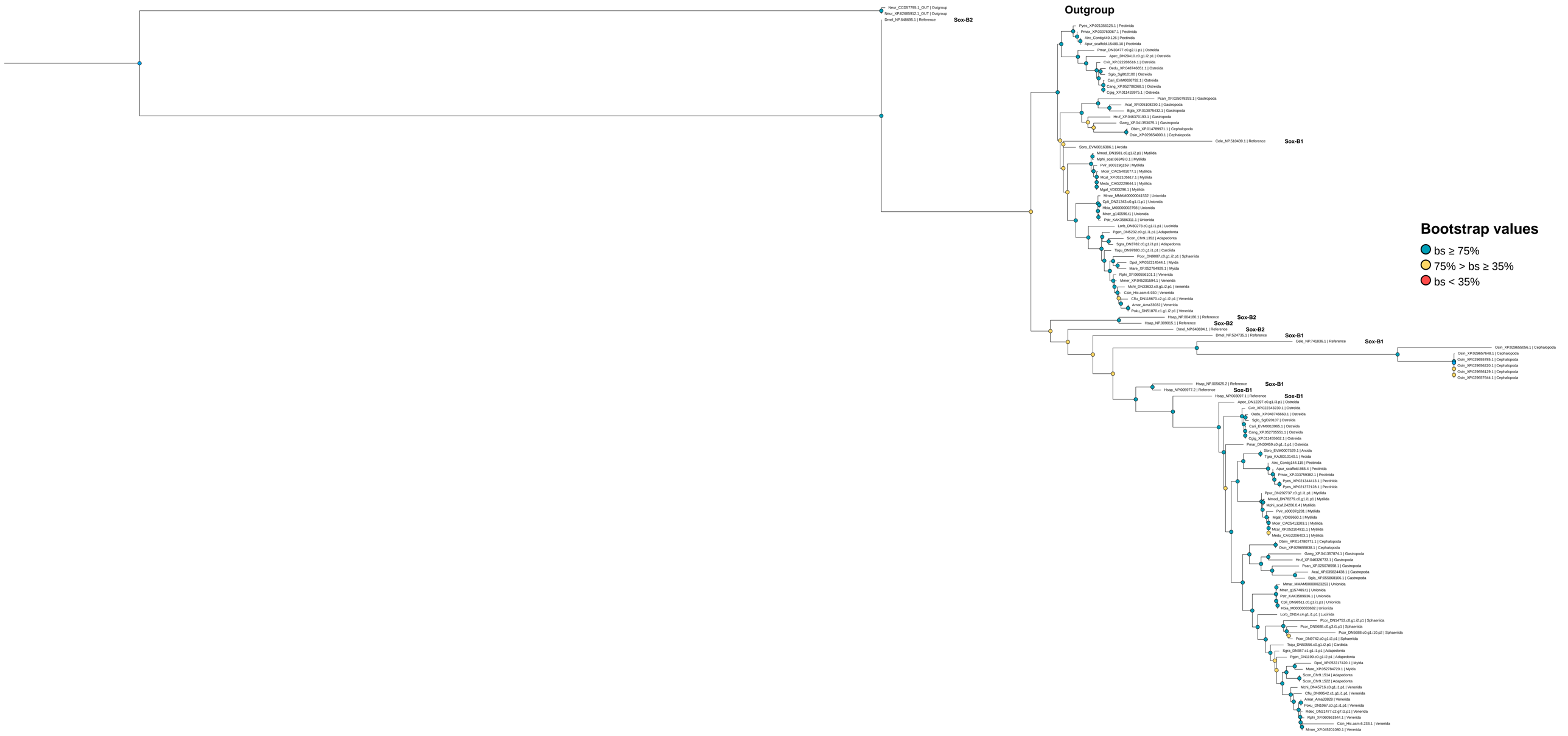

Supplement: Supplementary file 13 — Figure S13: ML phylogenetic tree of Sox‐B1 and Sox‐B2 genes in mollusc and reference species. For each tip, the species ID, the gene ID and the taxonomic information are provided. Taxonomical information is replaced by ‘Reference’ if the sequence was used to assess orthology. Species ID can be found in Table S1. Bootstrap values are shown for each node as points colour‐coded by intervals. Annotations of reference genes are reported next to the relative tip. [file MEC-34-e70103-s004.pdf]

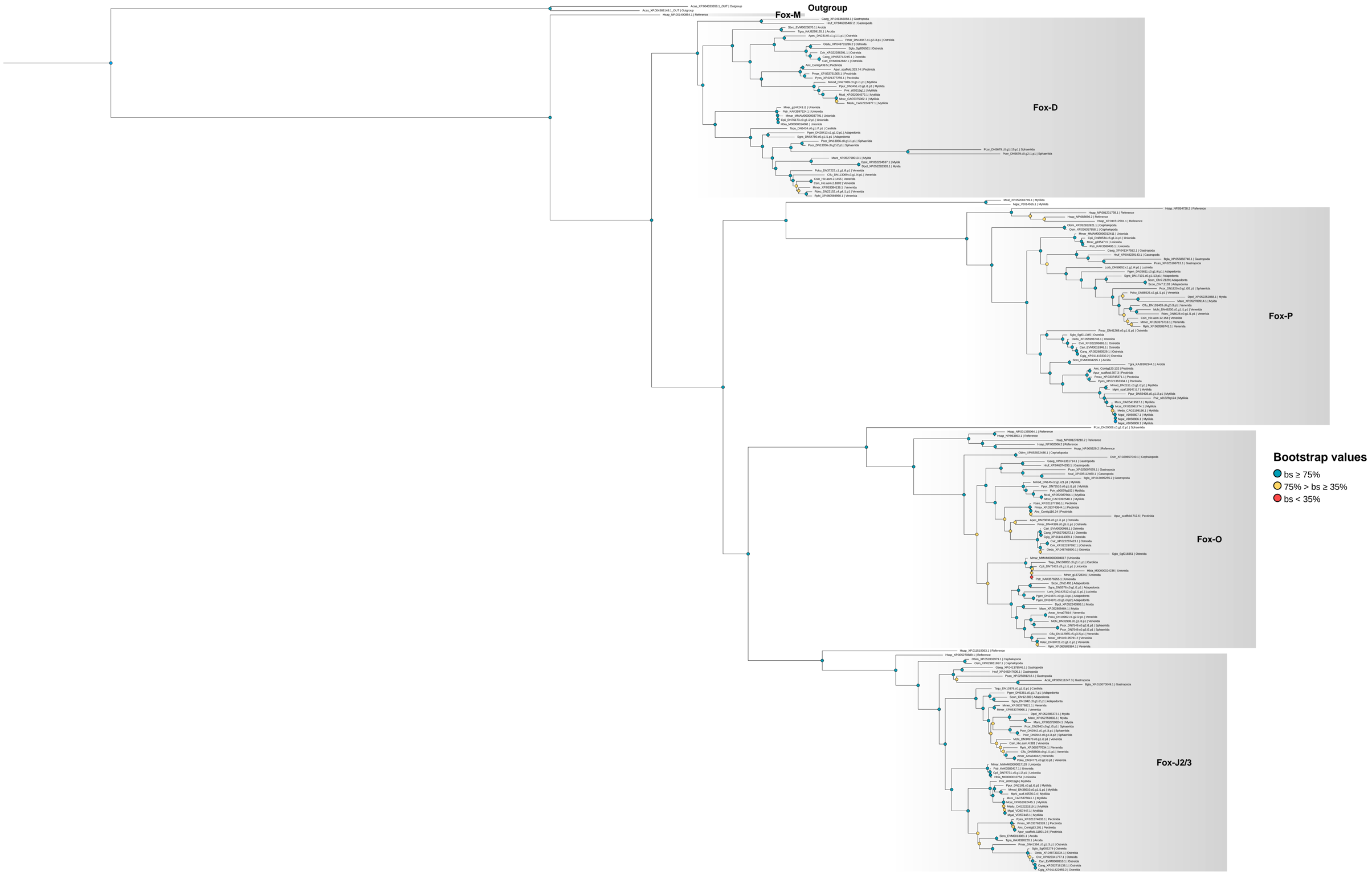

Supplement: Supplementary file 14 — Figure S14: ML phylogenetic tree of Fox‐J2, Fox‐M, Fox‐O and Fox‐P genes in mollusc and reference species. For each tip, the species ID, the gene ID and the taxonomic information are provided. Taxonomical information is replaced by ‘Reference’ if the sequence was used to assess orthology. Species ID can be found in Table S1. Bootstrap values are shown for each node as points colour‐coded by intervals. Major gene groups, as in Figures S2 and S3 are indicated with shaded rectangles and labels on the right of the tree. [file MEC-34-e70103-s006.pdf]

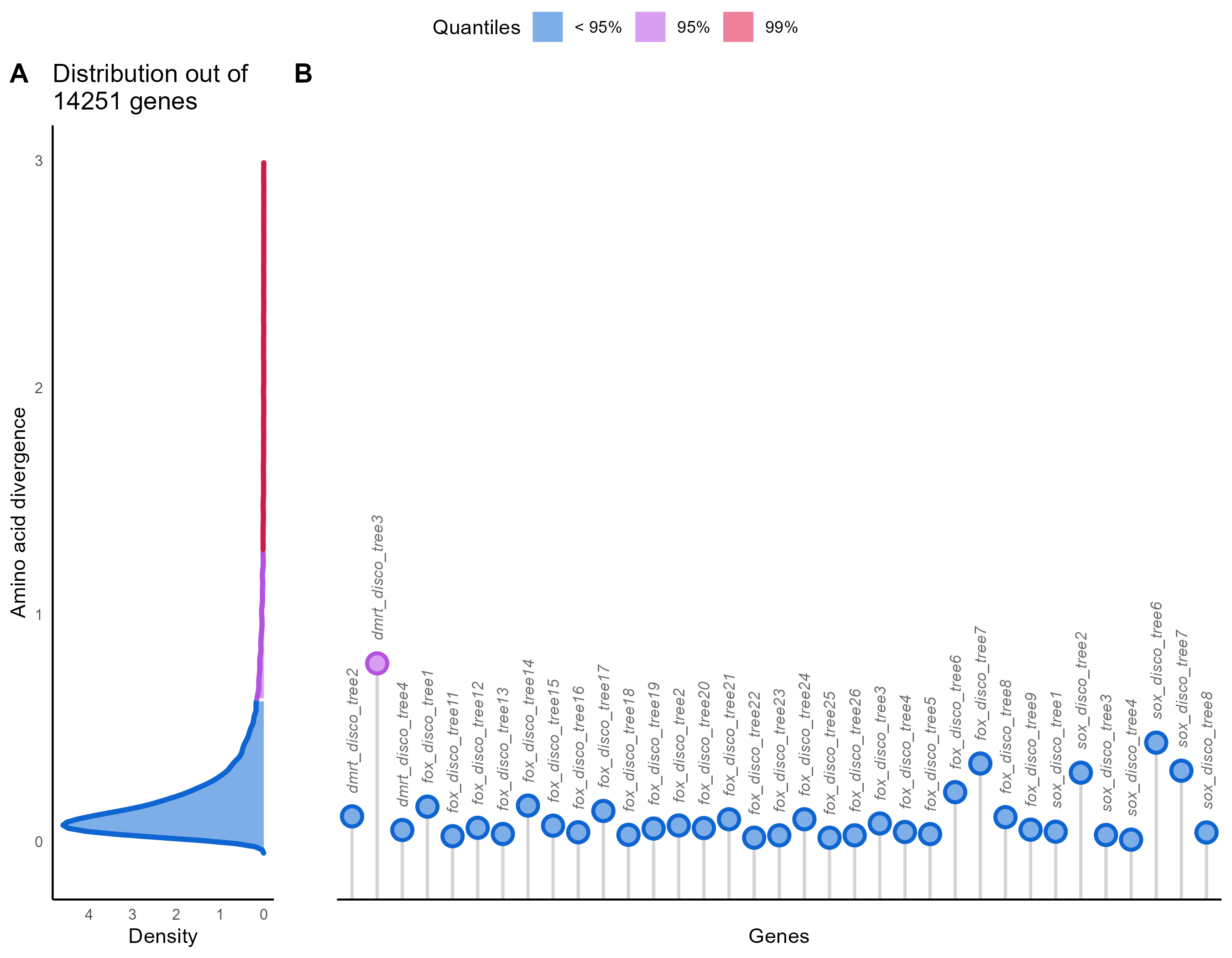

Supplement: Supplementary file 16 — Figure S16: Distribution of amino acid sequence divergence (AASD) of single‐copy orthogroups in Crassostrea gigas , Crassostrea angulata , Crassostrea ariakensis and Crassostrea virginica (A), including DSFG (B). The distribution of AASD in Crassostrea has been computed on the median values of pairwise distances of over 14 k single‐copy orthogroups (SCOs). Circle heights of DSFGs show the median value of their AASD. Dmrt‐1L genes are indicated as ‘dmrt_disco_tree3’. [file MEC-34-e70103-s001.png]
