# Supplementary material for: Comparative Genomics of Sex‐Determination‐Related Genes Reveals Shared Evolutionary Patterns Between Bivalves and Mammals, but Not Fruit Flies
Source: Mol Ecol. 2025 Sep 21;34(20):e70103. doi: 10.1111/mec.70103 (PMC12530298; doi:10.1111/mec.70103)
Supplement: Supplementary file 2 — Figure S2: ML phylogenetic tree of the Sox gene family in molluscs, including the Possvm orthology inference. For each tip, the species ID, the gene ID, the taxonomic information and the annotation as returned by the Possvm algorithm, are provided. Taxonomical information is replaced by ‘Reference’ if the sequence was used to assess orthology. Species ID can be found in Table S1. Bootstrap values are shown for each node as points colour‐coded by intervals. Major gene groups, as in Figures 2 and 3 are indicated with shaded rectangles and labels on the right of the tree. [file MEC-34-e70103-s009.pdf]

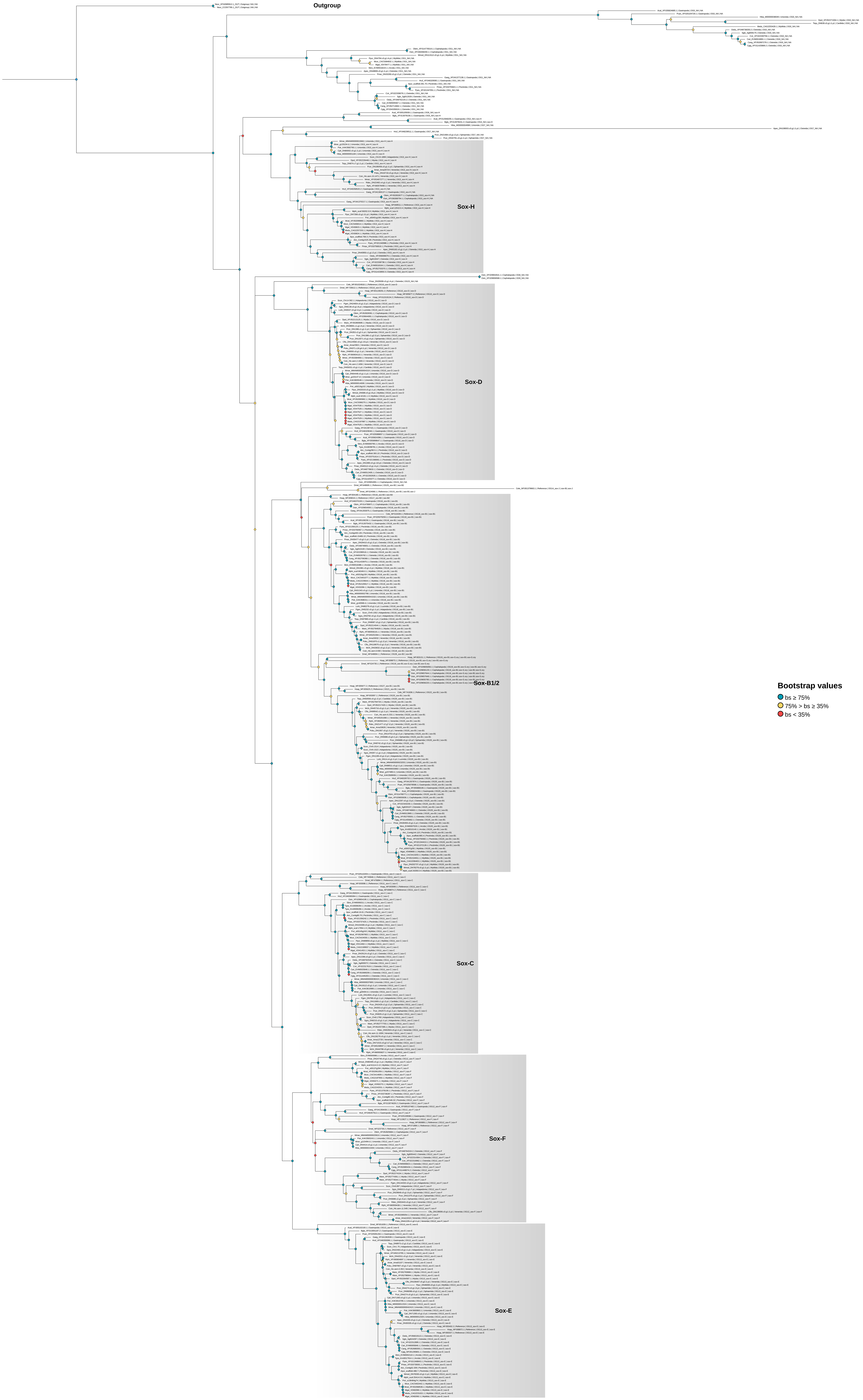
Outgroup
Sox-H
Sox-D
Sox-B1/2
Sox-C
Sox-F
Sox-E
Bootstrap values
bs ≥ 75%
75% > bs ≥ 35%
bs < 35%
