# Supplementary material for: Comparative Genomics of Sex‐Determination‐Related Genes Reveals Shared Evolutionary Patterns Between Bivalves and Mammals, but Not Fruit Flies
Source: Mol Ecol. 2025 Sep 21;34(20):e70103. doi: 10.1111/mec.70103 (PMC12530298; doi:10.1111/mec.70103)
Supplement: Supplementary file 7 — Figure S7: ML phylogenetic tree of the Sox gene family in mammals, including the Possvm orthology inference. For each tip, the species ID, the gene ID, the taxonomic information and the annotation as returned by the Possvm algorithm, are provided. Species ID can be found in Table S4. Bootstrap values are shown for each node as points colour‐coded by intervals. Major gene groups, as in Figure S5, are indicated with shaded rectangles and labels on the right of the tree. [file MEC-34-e70103-s013.pdf]

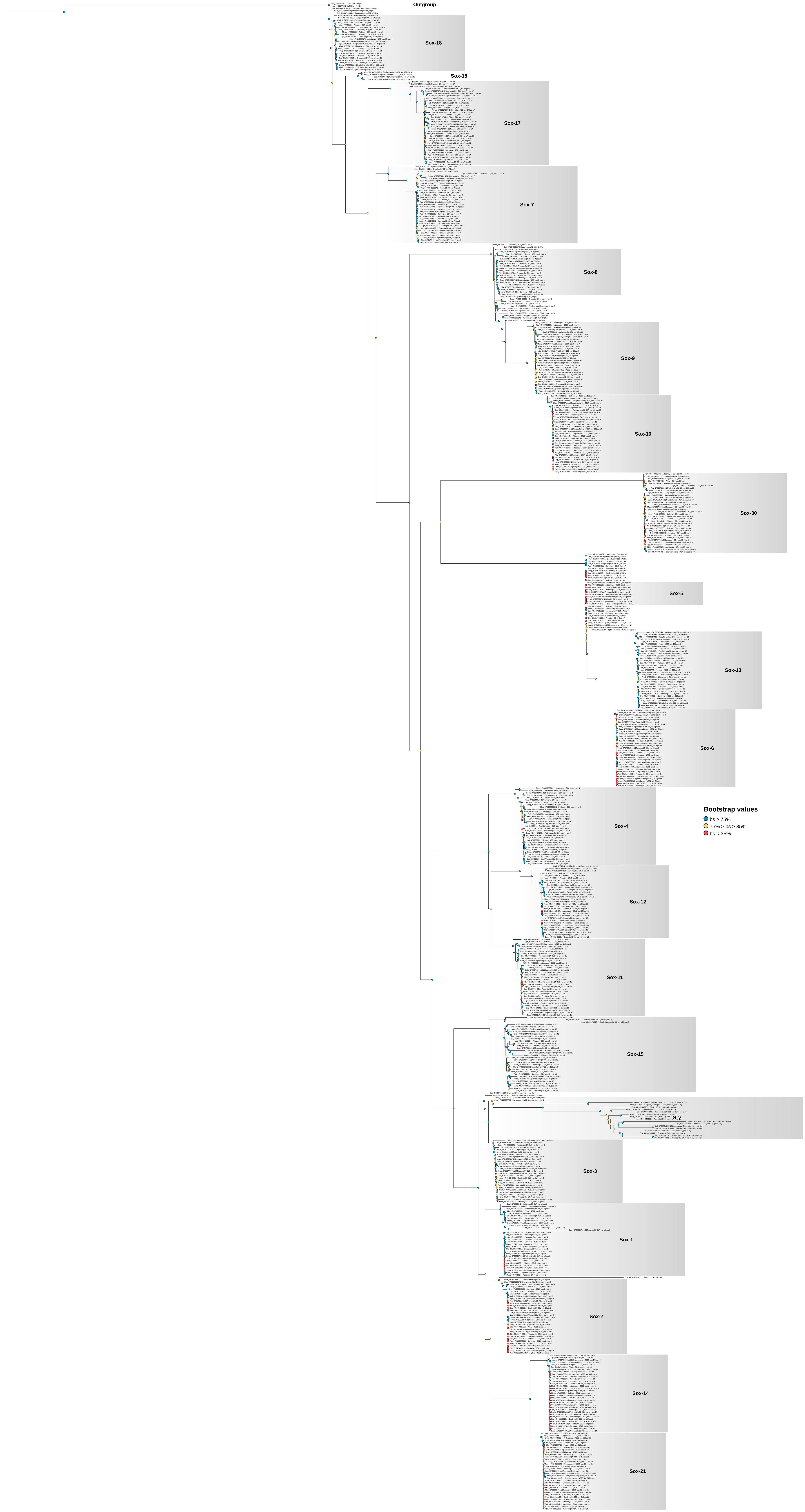

Outgroup
Sox-18
Sox-18
Sox-17
Sox-7
Sox-8
Sox-9
Sox-10
Sox-30
Sox-5
Sox-13
Sox-6
Bootstrap values
bs ≥ 75%
75% > bs ≥ 35%
bs < 35%
Sox-4
Sox-12
Sox-11
Sox-15
Sry
Sox-3
Sox-1
Sox-2
Sox-14
Sox-21
