# Supplementary material for: Comparative Genomics of Sex‐Determination‐Related Genes Reveals Shared Evolutionary Patterns Between Bivalves and Mammals, but Not Fruit Flies
Source: Mol Ecol. 2025 Sep 21;34(20):e70103. doi: 10.1111/mec.70103 (PMC12530298; doi:10.1111/mec.70103)
Supplement: Supplementary file 15 — Figure S15: ML phylogenetic tree of the Fox gene family in bivalves and the sea urchin Strongylocentrotus purpuratus (Spur). For each tip, the species ID, the gene ID, and the taxonomic information are provided. Taxonomical information is replaced by ‘Reference’ if the sequence was used to assess orthology. Species ID can be found in Table S1. S. purpuratus genes are those given by Tu et al. (2006). Bootstrap values are shown for each node as points colour‐coded by intervals. Major gene groups, as in Figure S4, are indicated with shaded rectangles and labels on the right of the tree. [file MEC-34-e70103-s015.pdf]

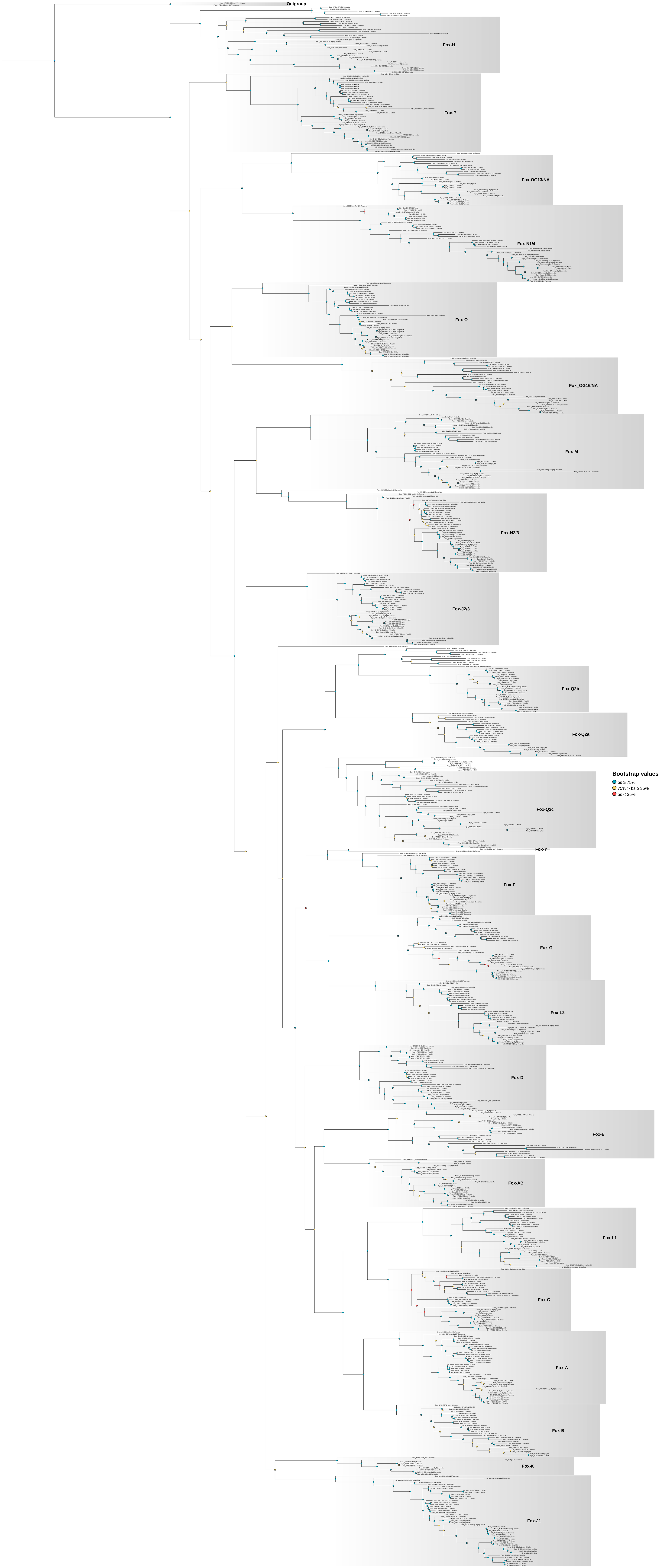
Outgroup
Fox-H
Fox-P
Fox-OG13/NA
Fox-N1/4
Fox-O
Fox_OG16/NA
Fox-M
Fox-N2/3
Fox-J2/3
Fox-Q2b
Fox-Q2a
Fox-Q2c
Fox-Y
Fox-F
Fox-G
Fox-L2
Fox-D
Fox-E
Fox-AB
Fox-L1
Fox-C
Fox-A
Fox-B
Fox-K
Fox-J1
Bootstrap values
bs ≥ 75%
75% > bs ≥ 35%
bs < 35%
